# Analysis of the Putative Nucleoporin POM33 in the Filamentous Fungus *Sordaria macrospora*

**DOI:** 10.3390/jof7090682

**Published:** 2021-08-24

**Authors:** Anika Groth, Kerstin Schmitt, Oliver Valerius, Britta Herzog, Stefanie Pöggeler

**Affiliations:** 1 Department of Genetics of Eukaryotic Microorganisms, Institute of Microbiology and Genetics, Georg-August-University of Göttingen, Grisebachstr. 8, 37077 Göttingen, Germany; anika.gibron@uni-goettingen.de (A.G.); bherzog@gwdg.de (B.H.); 2 Department of Molecular Microbiology and Genetics, Service Unit LCMS Protein Analytics, Institute of Microbiology and Genetics, Georg-August-University of Göttingen, Grisebachstr. 8, 37077 Göttingen, Germany; kschmit1@gwdg.de (K.S.); ovaleri@gwdg.de (O.V.)

**Keywords:** STRIPAK complex, POM33, transmembrane nucleoporins, nuclear envelope, endoplasmic reticulum, *Sordaria macrospora*

## Abstract

In the filamentous fungus *Sordaria macrospora* (Sm), the STRIPAK complex is required for vegetative growth, fruiting-body development and hyphal fusion. The SmSTRIPAK core consists of the striatin homolog PRO11, the scaffolding subunit of phosphatase PP2A, SmPP2AA, and its catalytic subunit SmPP2Ac1. Among other STRIPAK proteins, the recently identified coiled-coil protein SCI1 was demonstrated to co-localize around the nucleus. Pulldown experiments with SCI identified the transmembrane nucleoporin (TM Nup) SmPOM33 as a potential nuclear-anchor of SmSTRIPAK. Localization studies revealed that SmPOM33 partially localizes to the nuclear envelope (NE), but mainly to the endoplasmic reticulum (ER). We succeeded to generate a Δpom33 deletion mutant by homologous recombination in a new *S. macrospora* Δku80 recipient strain, which is defective in non-homologous end joining. Deletion of *Smpom33* did neither impair vegetative growth nor sexual development. In pulldown experiments of SmPOM33 followed by LC/MS analysis, ER-membrane proteins involved in ER morphology, protein translocation, glycosylation, sterol biosynthesis and Ca^2+^-transport were significantly enriched. Data are available via ProteomeXchange with identifier PXD026253. Although no SmSTRIPAK components were identified as putative interaction partners, it cannot be excluded that SmPOM33 is involved in temporarily anchoring the SmSTRIPAK to the NE or other sites in the cell.

## 1. Introduction

**Figure 1:**
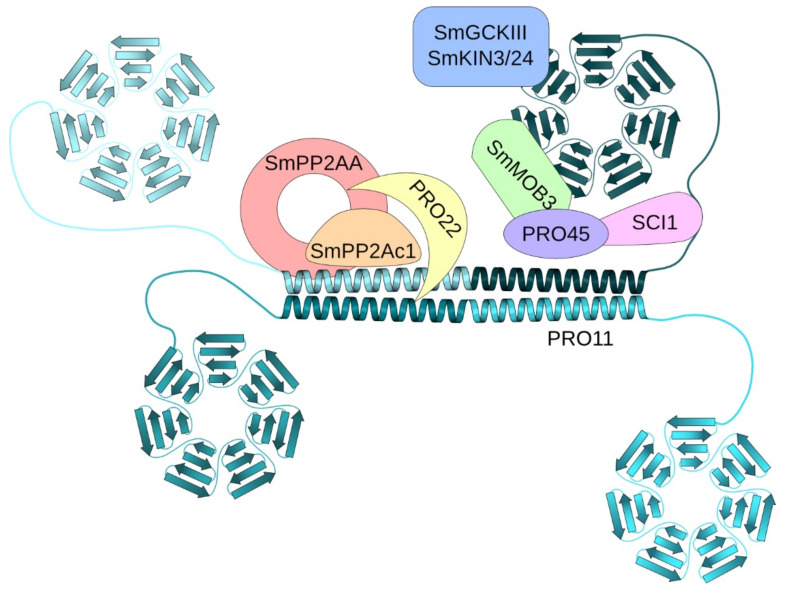
Schematic illustration of the SmSTRIPAK complex. According to structural data of the human STRIPAK complex [15] and data from *S. macrospora* [6,7,8,9,10,11,12,13,14], the SmSTRIPAK complex consists of a tetramer of the scaffolding human striatin homolog PRO11 harboring an N-terminal coiled-coil domain (cyan-shaded helices) and C-terminal WD40 repeats (cyan-shaded beta-propeller). Further components are the STRIP1/2 homolog PRO22 (yellow), SmMOB3 (green), the scaffolding subunit of phosphatase PP2A, SmPP2AA (red), and its catalytic subunit SmPP2Ac1 (orange) and recruited GCKIII kinases SmKIN3/24 (blue). Moreover, the SLMAP homolog PRO45 (purple) and the coiled-coil protein SCI1 (pink) are key subunits of the SmSTRIPAK.

The striatin-interacting phosphatase and kinase (STRIPAK) complex is a conserved multiprotein complex involved in diverse cell-signaling pathways like cell growth and -fusion, vesicular traffic, endocytosis and even apoptosis in animals and fungi [1,2]. The main components of STRIPAK complexes are striatin scaffolding proteins [3]. The coprophilic ascomycete *Sordaria macrospora* (Sm) is a fungal model organism to study complex fruiting-body development [4,5]. In *S. macrospora*, SmSTRIPAK plays an important role in vegetative growth, hyphal fusion and sexual development. The major scaffold protein of the SmSTRIPAK complex is the human striatin homolog PRO11 [6,7,8]. In addition, the SmSTRIPAK complex consists of the STRIP1/2 homolog PRO22, SmMOB3, the scaffolding subunit of phosphatase PP2A, SmPP2AA, and its catalytic subunit SmPP2Ac1 as well as recruited GCKIII kinases SmKIN24 and SmKIN3 [6,9,10,11,12,13]. Moreover, the SLMAP homolog PRO45 and the coiled-coil protein SCI1 were identified as key subunits of the SmSTRIPAK [9,14]. Recently, the cryo-EM structure of the human STRIPAK core was determined. There, the coiled-coil domains of striatin form a homotetrameric platform for the interaction with one copy of PP2AA and PP2Ac, STRIP1 and MOB4 [15]. A similar structure can be assumed for SmSTRIPAK (Figure 1). Fluorescence microscopic investigations revealed that SCI1 and PRO11 both co-localize around the nucleus [14]. In addition, the STRIPAK components SLMAP homolog PRO45 and SmMOB3 were localized to the nuclear envelope (NE), endoplasmic reticulum (ER), and mitochondria [9].

In pulldown experiments coupled to LC/MS analysis, the putative TM Nup SmPOM33 had previously been identified as putative interaction partner of the SmSTRIPAK component SCI1 [14]. Together with this result and the observation that SCI1 and other STRIPAK components co-localize around the nucleus, we addressed the question if SmPOM33 might be anchoring the SmSTRIPAK to the nucleus. The nucleus is surrounded by a double-lipid bilayer comprising the inner nuclear membrane (INM) that merges into the outer nuclear membrane (ONM) at sites of nuclear pores and is continuous with the endoplasmic reticulum forming the nuclear envelope [16]. The ER plays an important role in many cellular processes, like protein synthesis and modification, lipid synthesis and Ca^2+^-regulation, homeostasis and secretion [17]. It consists of peripheral sheet-like cisternae and a network of interconnected ER tubules [18]. Its morphology is divided into the rough ER (RER) with membrane-bound ribosomes and the ribosome-absent smooth ER (SER) [19]. The tubules of the ER undergo continuous fusion and partitioning events to establish three-way junctions showing that the ER network is highly dynamic [17,18,20,21]. At the fusion sites of the INM and the ONM, nuclear-pore complexes (NPCs) are incorporated. NPCs are large protein complexes that form channels to mediate nucleocytoplasmic exchange [22]. The assembly of NPCs-consisting proteins, termed nucleoporins (Nups), can occur postmitotic or *de novo*. In case of postmitotic NPC biogenesis, the NE reforms from cortical ER (cER) into a pore that is stabilized by pore membrane proteins (Poms) so that Nups are recruited [23,24,25,26]. During *de novo* NPC assembly, the pore is formed by fusion of the double membrane system of the NE. For this process, the conserved protein Ndc1 is the only essential transmembrane Nup (TM Nup) so far known [27,28,29]. Besides Ndc1, three other TM Nups Pom33/Tts1/TMEM33 assist in NPC assembly [30,31,32].

In this study, we performed fluorescence microscopy, generated a *Smpom33* deletion mutant ΔSmpom33 and did pulldown experiments coupled to LC/MS analysis to examine if we can pull down SCI1 or other SmSTRIPAK components vice versa. We showed that SmPOM33 localizes at the ER and NE. Pulldown experiments with a tagged version of SmPOM33 identified no SmSTRIPAK components but revealed ER-membrane proteins involved in ER morphology, protein translocation, glycosylation, sterol biosynthesis and Ca^2+^-transport as putative interaction partners.

## 2. Materials and Methods

### 2.1. Strains, Media and Growth Conditions

All strains used in this study are presented in Table 1. For cloning and propagation of recombinant plasmids, *Escherichia coli* MACH1 (Thermo Fisher Scientific, C862003, Waltham, MA, USA) was used in standard culture conditions [33]. To generate recombinant plasmids, homologous recombination (HR) was performed using *Saccharomyces cerevisiae* strain PJ69-4A [34,35] and positive clones were selected for uracil prototrophy. *S. macrospora* strains were transformed with the recombinant plasmids (Table S1 in Supplementary Materials) as described in the standard protocol [36,37]. Positive transformants were selected on media containing nourseothricin-dihydrogen sulfate (50 µg/mL, nat) (Jena Bioscience GmbH, AB-102XL, Jena, Germany) and/ or hygromycin B (110 U/mL, hyg) (Merck, 4400051-10MU, Kenilworth, NJ, USA). *S. macrospora* strains were grown on liquid or solid biomalt maize medium (BMM) or Sordaria Westergaard (SWG) fructification medium at 27 °C under continuous light conditions [38,39,40]. Crosses of different *S. macrospora* strains were performed as described previously [13]. For phenotypic analysis under different stress conditions, the temperature was changed to 16 °C and 37 °C or SWG medium was supplemented with 0.1 M NaCl, 0.01% H_2_O_2_, 100 µg/mL Calcofluor White (CFW), 0.2 µg/mL Tunicamycin (TM) or 1.5 mM DTT. For microscopic analyses, *S. macrospora* strains were grown under continuous light at 27 °C on solid SWG or selective BMM medium covered with Cellophane or on BMM-agar slides. For the agar slides, two sterile toothpicks were placed in an empty petri dish, a glass slide was laid on top and covered with fresh BMM media (2% agar and 50 mL clear liquid BMM). After inoculating the BMM-covered slide with a piece of fungal mycelia from an agar plate, liquid BMM was poured carefully into the petri dish slightly covering the slide to increase the humidity. Dishes were incubated at 27 °C over night. Dependent on the developmental stage analyzed, the growth period differed between 24 h or 2–10 days.

### 2.2. Generation of Plasmids

All plasmids and primers (Sigma-Aldrich Chemie GmbH Taufkirchen, Germany) used in this study are shown in Tables S1 and S2 in Supplementary Materials. Plasmids were constructed via HR in *S. cerevisiae* [34] or the Golden Gate (GG) cloning system [45]. For p5′sci1-egfp_hyg, the *S. macrospora sci1* native promotor and ORF and the *trpC* terminator (*TtrpC*) of *Aspergillus nidulans* were amplified from plasmid p5′sci1gfp_nat [14] with the primer pair 5′sci1-f/pRS426GFPrev. The fragment was cloned into *Xho*I-linearized vector pRS-hyg [6] via HR in the *S. cerevisiae* strain PJ69-4A [35]. To generate the plasmid p5′pom33-egfp, plasmid p5′sci1gfp_nat [14] served as template and was hydrolyzed with *Bgl*II to cut out the *sci1* promoter and ORF. The *S. macrospora pom33* promoter (*Ppom33*) and the *pom33* ORF were amplified from *S. macrospora* wildtype (DSM997) (wt) gDNA using primer pair Smpom33-f/Smpom33-egfp-r. The resulting fragment was integrated into *Bgl*II-digested p5′sci1gfp_nat via HR in *S. cerevisiae*. For plasmids p5′pom33-TagRFP-T_hyg/_nat, a fragment comprised of the *pom33* promoter and *pom33* ORF was amplified with primer pair Smpom33-f/Smpom33-RFP-r from wt gDNA and a fragment comprised of *TagRFP-T* and the *TtrpC* terminator of *A. nidulans* was amplified with primer pair RFP-f/pRS426GFPrev from pTagRFP-T_nat [44]. Both fragments were integrated into *Xho*I-linearized pRS-nat [46] or pRS-hyg [6] via HR in *S. cerevisiae*.

For the construction of the plasmid pRSku80::hph, the primer pair ku80-5f/ku80-5-hyg-r was used to amplify the 5′ flanking region (1021 bp) of the *ku80* gene. For amplification of the *ku80* 3′ flanking region (1010 bp) primer pair ku80-3-hyg-f/ku80-3r was used. The flanking regions were amplified from *S. macrospora* gDNA and contained an 29-bp overhang to pRS426 vector [47] and the *hph*-cassette, respectively. The *hph*-resistance cassette (1385 bp) was amplified from plasmid pCB1003 [48] with primer hph-f and hph-r. The three PCR fragments were transformed with an *Xho*I-linearized pRS426 vector into the *S. cerevisiae* for HR.

To generate the knockout-plasmid pPom33-KO, the Golden Gate cloning system according to [45] was used. The 5′ (726 bp) and 3′ (1021 bp) flanking regions of the *pom33* gene were amplified from *S. macrospora* wt gDNA with primer pairs Pom33-GG-ko-5f/Pom33-GG-ko-5r and Pom33-GG-ko-3f/Pom33-GG-ko-3r, respectively. Together with donor vector pGG-nat1 and the destination vector pDest-Amp, the fragments were cloned via the Golden Gate procedure [45].

DNA sequencing of the plasmids was performed by Seqlab Sequence Service Laboratories GmbH (Göttingen, Germany).

### 2.3. Generation of the Knockout Strains Δku80 and Δpom33

For the deletion of the *S. macrospora ku80* gene, the deletion cassette was amplified from pRSku80::hph with primer pair ku80-5f/ku80-3r and transformed into the nourseothricin resistant *S. macrospora* strain Δku70 [42]. To eliminate the Δku70 background hygromycin/nourseothricin resistant strains were crossed with the spore color mutant fus1-1 (S23442) [41]. Single-spore isolates were selected on BMM medium supplemented with hygromycin. The single-spore mutant was named Δku80 and the deletion of *ku80* was verified by PCR and Southern blot analyses. To verify the presence of the *hph*-cassette at the desired gene locus, primer pair ku80-ko-v5f/tC1 (1181 bp) and h3/ku80-r (1152 bp), respectively, were used. Absence of the *ku80* gene in the generated Δku80 strain was verified with primer pairs ku80-ko-v5f/ku80-1r (1581 bp) and ku80-1f/ku80-1r (484 bp). For Southern hybridization, 30 µg of wt and Δku80 gDNA were digested with *Aat*II and *Bgl*II. Electrophoresis of digested gDNA was performed on an 0.8% agarose (Biozym Scientific GmbH, Hessisch Oldendorf, Germany) gel. A capillary blot with nylon membrane (GE Healthcare, Amersham RPN303B, Boston, MA, USA) was performed at room temperature (RT) overnight. The corresponding probes for the 5′- and 3′- flanking regions were PCR amplified with primer pair ku80-ko-v5f/ku80-ko-v5r and ku80-ko-v3f/ku80-r, respectively.

To delete the *S. macrospora pom33* gene, the knockout plasmid pPom33-KO was used as template to amplify the 2645 bp deletion cassette with the primer pair GG_KO_fw/GG_KO_rv, containing the 5′- and 3′- flanking regions of *pom33* and the *nat* cassette. After PCR-clean-up of the amplicon, the *S. macrospora* Δku80 strain was transformed with the deletion cassette to replace the *pom33* ORF with the *nat*-cassette. Primary transformants were crossed with the color-spore mutant fus1-1 [41] and single-spore isolates of Δpom33 carrying nourseothricin resistance were selected [13]. Verification of the absence of the *pom33* gene and integration of the *nat*-cassette at the desired locus was performed with primer pairs Pom33-v2f/tC1 (948 bp) and Smpom33-vORF3-f/Pom33-v2r (1339 bp), respectively. To check the presence of the *hph*-cassette in Δku80 and the *ku80* gene in Δpom33 after crossing, primer pairs ku80-ko-v5f/tC1 (1176 bp) and ku80-ko-v5f/ku80-1r (1580 bp), respectively, were used. Deletion of *Smpom33* was further verified via Southern hybridization. For this, 30 µg of wt and Δpom33 gDNA were hydrolyzed with *Pst*I and separation of digested gDNA was done on an 0.8% agarose (Biozym Scientific GmbH, Hessisch Oldendorf, Germany) gel. A capillary blot with nylon membrane (GE Healthcare, Amersham RPN303B, Boston, MA, USA) was performed at RT overnight. The 726-bp *pom33* probe was amplified from *S. macrospora* wt gDNA with primer pair Pom33-GG-ko-5f/Pom33-GG-ko-5r.

Labeling of probes for Southern blot experiments was done using the Amersham AlkPhos Direct Labelling and Detection Kit (GE Healthcare, Amersham RPN3680, Boston, MA, USA). Detection was performed according to the manufacturer’s manual. Signals were visualized on X-ray films (Amersham Hyperfilm^TM^ ECL, Marlborough, MA, USA) using an “Optimax X-ray film processor” (PROTEC GmbH & Co. KG, Oberstenfeld, Germany).

### 2.4. Light and Fluorescence Microscopy

To investigate vegetative hyphae and sexual structures, *S. macrospora* strains were grown on solid SWG medium covered with a piece of cellophane (0.5 cm × 0.5 cm) over 2 to 9 days and documented with an AxioImage M1 microscope (Zeiss, Jena, Germany) using differential-interference contrast (DIC) or a VHX-500F Digital Microscope (Keyence, Neu-Isenburg, Germany). Images were captured with a Photometrix CoolSNAP HQ camera (Roper Scientific, Photometrics, Tucson, AZ, USA). Image processing was done using ZEISS ZEN Digital Imaging (version 2.3; Zeiss, Jena, Germany) and the Affinity Publisher software (version 1.9.2.1035, Serif (Europe) Ltd., Nottingham, UK, https://affinity.serif.com/de/publisher/; accessed on 1 March 2021).

For fluorescence microscopic analyses, *S. macrospora* strains were grown on selective BMM medium on top of cellophane sheets or on BMM-covered glass slides for 24 h at 27 °C. For the detection of the EGFP signal Chroma filter set 49002 (exciter ET470/40x, ET525/50m, beamsplitter T495lpxr) and for TagRFP-T/tdTomato Chroma filter set 49005 (exciter ET545/30x, emitter ET620/60m and beamsplitter T570LP) was used.

### 2.5. Protein Sample Preparation and Western Blot Hybridization

To extract proteins from fungal mycelium, *S. macrospora* strains were cultivated in liquid BMM medium and grown for 3 days at 27 °C. The mycelium was harvested, dried and grounded in liquid nitrogen. After adding 520 µL lysis buffer (10 mM Tris-HCl pH 7.5, 150 mM NaCl, 0.5 mM EDTA pH 8.0, 1 mM PMSF, 2 mM DTT, 0.5% NP-40, 1} protease inhibitor cocktail IV (1tbl/50 mL, 04693132001, Mannheim, Germany), 1} PhosSTOP™ (1tbl/10 mL, Roche, 04906837001, Mannheim, Germany)) /g mycelium powder and ~200 µL glass beads (Ø 0.25–0.5 mm, Roth GmbH, A553.1, Karlsruhe, Germany), cells were lysed in the Tissue Lyser (Qiagen, Hilden, Germany) by 30 Hrz for 2 min. Subsequently, cells were separated from debris by centrifugation at 10,000 rpm for 15 min at 4 °C and were prepared for Western Blot analysis by adding 20 µL 4} NuPAGE^®^ LDS-SB (Thermo Fisher Scientific, NP0007, Waltham, MA, USA) and 4 µL 1 M DTT to 56 µL crude extract and subsequent heating for 10 min at 70 °C. A total of 25 µL of the samples were loaded on a 12% SDS gel. As a protein standard 5 µL of the Nippon Genetics Co. Europe blue star pre-stained protein marker (NIPPON Genetics Europe, MWP03, Düren, Germany) was used.

After separating the proteins they were transferred from the SDS-PAGE gel onto an Amersham^TM^ Protran^TM^ Nitrocellulose Blotting Membrane (GE Healthcare, RPN203B, Little Chalfont, UK) using 1× transfer buffer and a Mini Trans-Blot^®^ Cell device as described by the manufacturer (Bio-Rad Laboratories, Hercules, CA, USA) [49].

The nitrocellulose membrane was blocked with 5% (*w*/*v*) skim milk powder in 1} Tris-buffered saline supplemented with 0.05% Tween 20^®^ (TBST) for 1 h at RT. To detect antigen–antibody reaction, a primary EGFP (rat)- (1:4000, ChromoTek GmbH, 3h9-100, Planegg-Martinsried, Germany) or TagRFP-T (rabbit) -antibody (1:12,500, BioCat (Evrogen, Moscow, Russia), AB233-ev) solved in 5% skim milk/TBST was used and the membrane was incubated with the antibody solution over night at 4 °C. After removing the primary antibody, the membrane was washed three times with 1} TBST for 15 min and a horse-radish peroxidase (HRP) coupled secondary anti rat- or rabbit-antibody (1:5500, Thermo Fisher Scientific, 62-9520, Waltham, MA, USA; 1:5000, Thermo Fisher Scientific, G-21234, Waltham, MA, USA) was applied to the membrane for 1 h at room temperature. Enhanced chemiluminescence reaction was used to detect the HRP coupled antibodies using the Immobilon^TM^ Western HRP Substrate kit (Merck, WBKLS0500, Kenilworth, NJ, USA). Signals were visualized on X-ray films (Amersham Hyperfilm^TM^ ECL, Marlborough, MA, USA) using an “Optimax X-ray film processor” (PROTEC GmbH & Co. KG, Oberstenfeld, Germany).

### 2.6. Pulldown Experiments, LC/MS and Data Analysis

#### 2.6.1. Pulldown Experiments

In pulldown experiments, first three biological and in the second experiment additionally one technical replicate of the *S. macrospora* strains Δpom33::pom33-TagRFP-T^ect^ and as control wt::TagRFP-T^ect^ were used. Protein sample preparation was performed as described above but without NP-40 in the lysis buffer and with the first centrifugation step at 10,000 g for 20 min at 4 °C followed by a second centrifugation of the lysate at 10,000 g for 10 min at 4 °C. Here, the lysate of *S. macrospora* wt was used to dilute the control samples 1:1. For the pulldown, 1 mL of the lysate was incubated with 2 µL of primary TagRFP-T (rabbit) antibody (BioCat (Evrogen, Moscow, Russia), AB233-ev) for 2 h at 4 °C on a rotation wheel. Preparation of 50 µL Dynabeads^TM^ Protein G (Thermo Fisher Scientific, 10003D, Waltham, MA, USA) per ml lysate was performed by 30 s of vortexing and removing the supernatant after applying the magnet. The lysate-antibody solution was added to the beads and samples were rotated for 1 h at 4 °C. After applying the magnet and removing the supernatant, beads were washed twice with dilution buffer (lysis buffer without NP-40 and DTT). Beads were separated from the antibody-protein complex by adding 50 µL of 4} NuPAGE^®^ LDS-SB (Thermo Fisher Scientific, NP0007, Waltham, MA, USA) to get a 1} dilution and 5 µL 1 M DTT followed by heating for 10 min at 70 °C. A total of 25 µL of the control and samples were loaded on a 12% SDS gel and sample lanes were separated by lanes loaded with 5 µL of the Nippon Genetics Co. Europe blue star pre-stained protein marker (NIPPON Genetics Europe, MWP03, Düren, Germany). After separation of the proteins by SDS-PAGE, the gel was shaken for 30 min in fixing solution (10% acetic acid and 40% ethanol). Subsequently, the gel was washed with H_2_O for 10 min. Sample and control lanes were cut with a fresh scalpel into 4 small pieces and covered with ~200 µL HPLC-H_2_O for storing at 4 °C overnight in 1.5 mL Protein LoBind Tubes (0030108116, Eppendorf, Hamburg, Germany).

#### 2.6.2. Trypsin In-Gel Digest of Proteins and C18 Stage Tip Purification

Protein in-gel digestion with trypsin was performed according to [50]. After removing the water, 30 µL acetonitrile were added to the gel pieces and samples were incubated for 10 min at RT. Acetonitrile was taken off and gel pieces were dried for 10 min in the SpeedVac concentrator (Eppendorf concentrator 5301, Hamburg, Germany) at 50 °C. Further, 150 µL 10 mM DTT (in 100 mM NH_4_HCO_3_) was added and samples were incubated for 1 h at 56 °C. The DTT was removed and samples were incubated for 45 min at RT in the dark after 150 µL of 55 mM iodoacetamide (in 100 mM NH_4_HCO_3_) was added. To remove iodoacetamide, gel pieces were washed with 150 µL 100 mM NH_4_HCO_3_ and shaken for 10 min at RT. Next, the solution was discarded and 150 µL acetonitrile was added. Samples were shaken again for 10 min at RT before the solution was removed. The washing procedure was repeated once with 100 mM NH_4_HCO_3_ and acetonitrile. The gel pieces were dried for 10 min in the SpeedVac concentrator (Eppendorf concentrator 5301, Hamburg, Germany) and subsequently covered with sufficient trypsin digestion buffer (according to manufacturer’s information; SERVA Electrophoresis GmbH, 37283.01, Heidelberg, Germany). The samples were incubated on ice for 45 min and the excessive digestion buffer was removed. Gel pieces were covered with 25 mM NH_4_HCO_3_ pH 8.0 and incubated over night at 37 °C. After centrifugation at 13,000 rpm for 1 min at RT, the supernatant was transferred to a fresh 1.5 mL Protein LoBind Tube (0030108116, Eppendorf, Hamburg, Germany). Extraction of peptides was achieved by adding 20 mM NH_4_HCO_3_, shaking for 10 min at RT, centrifugation for 1 min at 13,000 rpm and subsequent addition 50% acetonitrile/ 5% formic acid to the gel pieces, shaking for 20 min at RT and centrifugation. The extraction with acetonitrile/ formic acid was repeated twice and all supernatants were collected and dried completely in the SpeedVac concentrator (Eppendorf concentrator 5301, Hamburg, Germany). For further purification, the peptide pellet was resolved in 20 µL fresh sample buffer (2% acetonitrile, 0.1% formic acid).

The C18 stage tip purification was performed as described previously [51]. The stage tips were equilibrated by the use of 100 µL methanol with 0.1% formic acid, followed by 100 µL 70% acetonitrile with 0.1% formic acid and two times 100 µL H_2_O with 0.1% formic acid. After adding the single solvent, the stage tips were centrifuged with the help of an adaptor in 2 mL reaction tubes at 13,000 rpm for 2 min and the flow through was discarded each time. The peptide samples were loaded on the C18 column, incubated for 5 min and centrifuged for 5 min at 4000 rpm. For a better yield, the flow through was reloaded and centrifuged under same conditions before the flow through was discarded. The column was washed twice with 100 µL H_2_O with 0.1% formic acid followed by centrifugation at 10,000 rpm for 2 min. To elute the peptides 60 µL 70% acetonitrile with 0.1% formic acid were added to the column and centrifuged at 4000 rpm for 5 min. The peptide solution was dried completely in the SpeedVac concentrator (Eppendorf concentrator 5301, Hamburg, Germany) and the pellet was resolved in 20 µL of sample buffer for LC/MS analysis.

#### 2.6.3. Liquid Chromatography—Mass Spectrometry (LC/MS) Analysis

For peptide separation, 2 µL of each sample were subjected to reverse phase liquid chromatography using an RSLCnano Ultimate 3000 system (Thermo Fisher Scientific, Waltham, MA, USA). Peptides were loaded on an Acclaim PepMap 100 pre-column (100 µm x 2 cm, C18, 5 µm, 100 Å; Thermo Fisher Scientific, Waltham, MA, USA) with 0.07% trifluoroacetic acid at a flow rate of 20 µL/min for 3 min. To separate the peptides analytically, an Acclaim PepMap RSLC column (75 µm x 50 cm, C18, 2 µm, 100 Å; Thermo Fisher Scientific, Waltham, MA, USA) with a flow rate of 300 nL/min was used. The solvent composition was gradually changed within 94 min from 96% Solvent A (0.1% formic acid) and 4% Solvent B (80% acetonitrile, 0.1% formic acid) to 10% Solvent B within 2 min, to 30% Solvent B within the next 58 min, to 45% Solvent B within the following 22 min, and to 90% Solvent B within the last 12 min of the gradient. All solvents and acids were prepared to have Optima grade for LC/MS (Thermo Fisher Scientific, Waltham, MA, USA). Nano-electrospray (nESI) using the Nanospray Flex Ion Source (Thermo Fisher Scientific, Waltham, MA, USA) at 1.5 kV (liquid junction) was used to on-line ionize eluting peptides, which were subsequently transferred into a Q Exactive HF mass spectrometer (Thermo Fisher Scientific, Waltham, MA, USA). At a resolution of 30,000, full scans in a mass range of 300 to 1650 m/z were recorded followed by data-dependent top 10 HCD fragmentation at a resolution of 15,000 (dynamic exclusion enabled). LC/MS method programming and data acquisition was performed using the XCalibur 4.0 software (Thermo Fisher Scientific, Waltham, MA, USA). Measurements were performed at the Service Unit LC/MS Protein Analytics of the Göttingen Center for Molecular Biosciences (GZMB) at the University of Göttingen, Germany.

### 2.7. Data Acquisition and Analysis

For protein identification, the LC/MS data were analyzed with the MaxQuant 1.6.0.16 software (Max-Planck-Institute of Biochemistry, Munich, Germany) [52]. The *S. macrospora*-specific peptide database Smacrospora_v03 [53], including the peptide sequence of free TagRFP-T, was used for database search with the Andromeda algorithm and the program’s default parameters. As the digestion mode trypsin/P was used and maximum missed cleavage sites were set to two. As fixed modifications carbamidomethylation of cysteins was considered and acetylation of the N-terminus and oxidation of methionine were set as variable modifications. Label free quantification (LFQ) was activated with a minimal ratio count of two to analyze the raw data. The decoy mode was revert with a false discovery rate of 0.01. Data evaluation and statistical analysis was performed with the Perseus 1.6.0.7 software (Max-Planck-Institute of Biochemistry, Munich, Germany) [54] and the online program Venny2.1 [55]. The protocol of the data processing is given in Table S3 in Supplementary Materials. BLAST searches of the best hits of identified proteins were conducted using the *Saccharomyces* Genome Database (SGD) (https://www.yeastgenome.org/blast-sgd; accessed on 12 April 2021) [56] and the UniProtKB BLAST tool (https://www.uniprot.org/blast/; accessed on 12 April 2021) [57] for fungi and *Homo sapiens*.

### 2.8. Protein Domain Analysis

Protein domains and position of the N- and C-terminus either in the lumen or the cytoplasm were predicted using the program InterProScan (https://www.ebi.ac.uk/interpro/search/sequence/; accessed on 15 March 2021) [58]. The coiled-coil motifs were predicted using NPS@: COILED-COILS PREDICTION (https://npsa.lyon.inserm.fr/cgi-bin/npsa_automat.pl?page=/NPSA/npsa_lupas.html; accessed on 15 March 2021) [59] and helices with the program NetSurfP—2.0 (https://services.healthtech.dtu.dk/service.php?NetSurfP-2.0; accessed on 15 March 2021) [60]. Design of the schematic illustration was performed in same relation to the amino acids indicated in the figure.

## 3. Results

### 3.1. S. macrospora POM33 Belongs to the Pom33/ TMEM33 Protein Family

In LC/MS analysis with *S. macrospora* STRIPAK complex protein SCI1 as bait, a protein encoded by SMAC_00774 had previously been identified as putative interaction partner and predicted to be a POM33 homolog via BLASTP analysis [14]. The 1219-bp coding region of the *pom33* gene from *S. macrospora* is interrupted by 3 introns and encodes a putative 286 aa transmembrane membrane protein of 33 kDa. In *S. cerevisiae*, *S. pombe* and *H. sapiens*, four transmembrane nucleoporins (TM Nups) are known so far, among them Pom33p in *S. cerevisiae*, TMEM33 in *H. sapiens* and Tts1p in *S. pombe* (Figure S1A in Supplementary Materials) [61]. BLASTP analysis with the SmPOM33 protein sequence revealed 31.71% sequence identity with the *H. sapiens* transmembrane protein 33 (TMEM33, NP_060596.2, e-value: 6e−07), 31.37% with *S. cerevisiae* nucleoporin Pom33 (Pom33p, NP_013077.1, e-value: 6e−46), 18.61% with its paralog Per33p (Pore and ER protein of 33 kDa) (Per33p, NP_013165.1, e-value: 1e−10), and 28.36% to the *S. pombe* tetra-spanning protein 1 (Tts1p, NP_596818.1, e-value: 4e−35). SmPOM33 is predicted to contain 4 transmembrane domains (TMD), one coiled-coil (CC) motif and 3 amphiphatic α-helices (Figure S1B in Supplementary Materials). Using the InterProScan program N- and C-terminus of SmPOM33 were predicted to point to the cytoplasm.

### 3.2. SmPOM33 Localizes at the Nuclear Envelope and the ER

**Figure 2:**
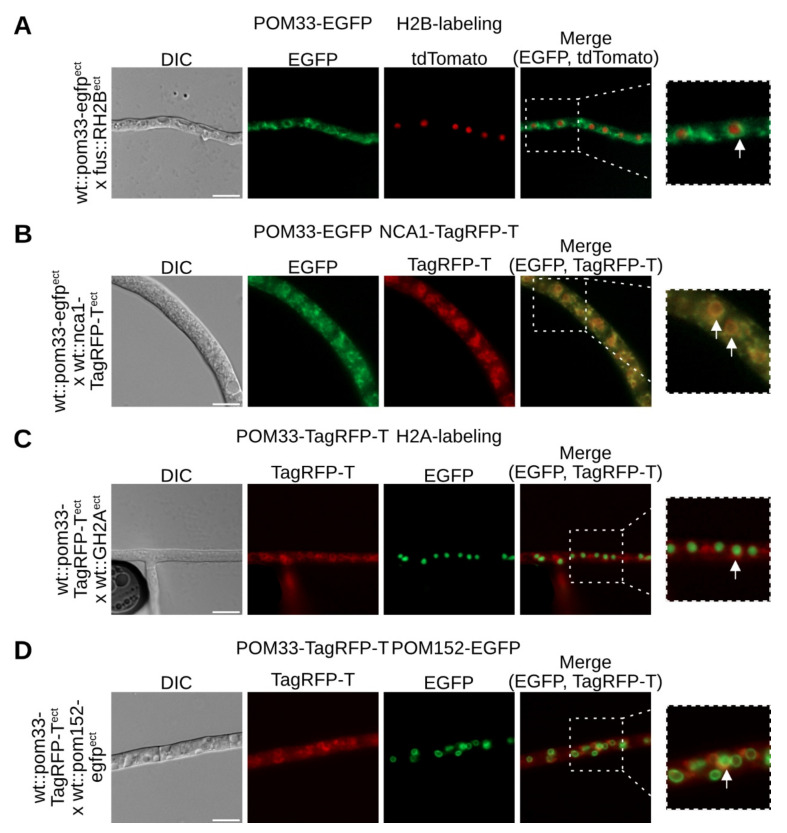
Co-localization of POM33 and marker proteins in *S. macrospora* wt using different fluorescence tags. *S. macrospora* wt expressing either POM33-EGFP or POM33-TagRFP-T was crossed with *S. macrospora* strains expressing marker proteins for the nucleus, endoplasmic reticulum (ER) and the nuclear envelope or the wt strain was co-transformed. Fluorescence microscopy was performed to visualize co-localization of the fusion proteins. (**A**) *S. macrospora* wt expressing POM33-EGFP was crossed with the fus1-1 strain expressing histone 2B labeled with tdTomato (RH2B). White arrow indicates localization of POM33-EGFP around the nucleus. (**B**) *S. macrospora* wt strains expressing POM33-EGFP and NCA1-TagRFP-T were crossed and co-localization of both proteins resulted in a yellow fluorescence signal is shown by the white arrows in the merged zoom-in picture. (**C**) *S. macrospora* wt expressing POM33-TagRFP-T and histone 2A tagged with EGFP (GH2A) were crossed to show localization of POM33 around the nucleus, independent of the fluorescence tag (white arrow in the merged zoom-in). (**D**) Partial co-localization of POM33-TagRFP-T with POM152-EGFP in *S. macrospora* wt (indicated by the white arrow). Scale bars = 10 µm. DIC, differential interference contrast. Detailed two-fold enlargements of the merge pictures are indicated with a dashed frame and shown at the right margin.

To determine the cellular localization of *S. macrospora* POM33, fluorescence microscopy was performed. SmPOM33 was C-terminally fused with either EGFP or TagRFP-T, to confirm the localization of POM33 independent of the tag. Both fusion constructs (POM33-EGFP and POM33-TagRFP-T) displayed a donut-like localization presumably around the nucleus that differs from the localization of free EGFP or TagRFP-T (Figure 2 and Figure S2 in Supplementary Materials). For localization of free EGFP or TagRFP-T, *S. macrospora* wt was transformed with plasmid p1783-1 [62] or pTagRFP-T_nat [44], respectively. To verify the localization of POM33 around the nucleus, we crossed the wildtype (wt) strain expressing POM33-EGFP with a fus1-1 (fus) strain expressing the histone 2B (H2B) fused to tdTomato (Figure 2A). The merged fluorescence image revealed the localization of POM33-EGFP as a fading ring around the red-labeled nucleus. To determine if POM33 also localizes to the endoplasmic reticulum (ER), *S. macrospora* wt strains expressing POM33-EGFP and the SERCA-type Ca^2+^-ATPase NCA1 tagged to TagRFP-T as a reporter protein for the ER and the NE, were crossed (Figure 2B). The resulting yellow fluorescence signal in the merged picture showed co-localization of POM33-EGFP with NCA1-TagRFP-T at the ER in the hyphae. To confirm the localization of POM33 around the nucleus independent of the fluorescence tag, a wt strain expressing POM33-TagRFP-T was crossed with a wt strain expressing histone 2A (H2A) fused to EGFP (Figure 2C). Here, the fluorescence signal of POM33-TagRFP-T was visible in ring-like structures around the green-labeled nuclei, supporting the localization of POM33 around the nucleus. Further, microscopic investigation of co-transformed wt strain expressing POM33-TagRFP-T and the TM Nup of the nuclear pore complex (NPC) POM152 C-terminally fused to EGFP, revealed partial co-localization of the fusion constructs with a more faded one of POM33-TagRFP-T as described above for POM33-EGFP (Figure 2D). To verify expression of POM33-TagRFP-T and POM33-EGFP, we performed Western blot experiments (Figure S3 in Supplementary Materials).

### 3.3. Deletion of pom33 Displays no Distinct Phenotype Compared to the Wildtype

For construction of knock-out strains of *S. macrospora* we usually use a Δku70 strain, which is impaired in the repair of DNA double-strand breaks and has been shown to be an ideal recipient strain for gene targeting experiments [42]. Primary transformants of *S. macrospora* are often heterokaryotic, therefore, we isolated single-spore isolates to segregate the wt and mutant alleles of *Smpom33* gene in a Δku70 background. Strains that were homokaryotic for the desired *Smpom33* deletion and carried the *ku70*-deletion were further crossed against color-spore mutant fus1-1. However, by conventional genetic analysis, we never succeeded in isolating hygromycin resistant spores without the Δku70 (nourseothricin-resistant) background, indicating that *Smpom33* and *ku70* are coupled genes. Non-homologous end joining involves the binding of the Ku heterodimer (Ku70/Ku80) at the ends of a DNA double-strand break (DSB) [63]. It has been previously demonstrated that homologous recombination in fungi is equally increased in Δku80 strains [64,65]. Therefore, we decided to generate a *S. macrospora* Δku80 deletion mutant. A BLAST search revealed that *SMAC_00164* encodes the homolog of the *N. crassa* KU80 protein. Deletion of the *ku80* gene was performed by homologous recombination of a *hph* deletion cassette in the Δku70 strain. After isolation of single-spore isolates and subsequent crosses to the spore-color mutant fus1-1, we succeeded in isolating a *hyg* resistant Δku80 strain without the Δku70 background. Deletion of the *ku80* gene was confirmed by PCR and Southern blot analysis (Figure S4 in Supplementary Materials). Similar to the Δku70 strain no impairment in vegetative growth or sexual development could be observed in the Δku80 strain, making it an ideal recipient strain for homologous recombination in *S. macrospora* as well (Figure S5 in Supplementary Materials).

**Figure 3:**
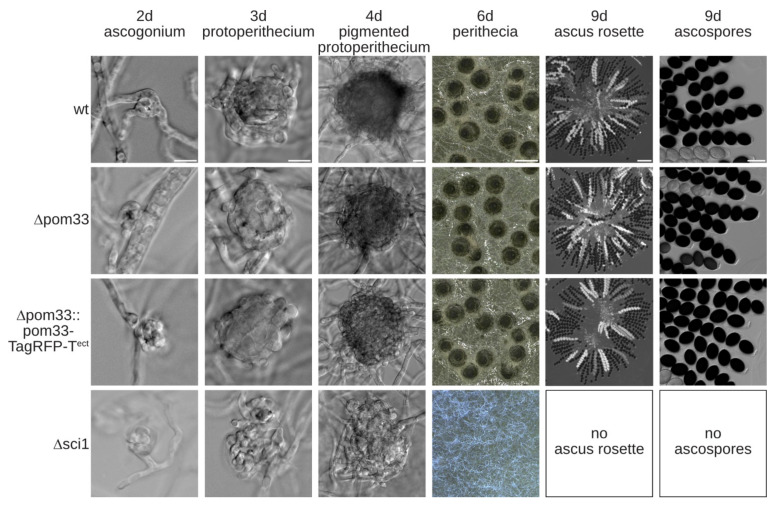
Sexual development in the wt, Δpom33, the complementation strain and Δsci1. Microscopic investigation of wt, Δpom33, the complementation strain (Δpom33::pom33-TagRFP-T^ect^) and Δsci1 grown over cellophane on SWG agar plates at 27 °C. Photographs were taken at indicated days. Scale bars from left to right: 10 µm; 10 µm; 10 µm; 0.5 mm; 100 µm and 25 µm.

We then used the Δku80 deletion strain to generate a Δpom33 deletion mutant of *S. macrospora*. To construct the Δpom33 strain, homologous recombination of a *nat* deletion cassette flanked by upstream and downstream regions of *pom33* was performed. Deletion of *pom33* was confirmed by PCR and Southern blot analysis (Figure S6 in Supplementary Materials). To examine the role of POM33 during sexual development, the life cycle of the deletion strain and a complementation strain, ectopically expressing the genomic version of *pom33* fused to *TagRFP-T* (Δpom33::pom33-TagRFP-T^ect^), were microscopically investigated together with the Δsci1 strain compared to the wt (Figure 3).

The wt, Δpom33 and the complemented strain completed the life cycle within 9 days and produced ascospores. The life cycle of *S. macrospora* starts with a germinating ascospore developing a vegetative mycelium. After 2–3 days, female gametangia (ascogonia) and after 3–4 days unpigmented fruiting-body precursors (protoperithecia) were produced that could be observed in all strains, respectively. These protoperithecia developed into melanin-pigmented large protoperithecia and after self-fertilization, karyogamy, meiosis and a postmeiotic-mitosis in the maturing perithecia, eight linear-arranged ascospores are present per ascus. Thus, the deletion mutant Δpom33 displayed no obvious phenotype in comparison to the wt (Figure 3). In contrast, STRIPAK deletion strain Δsci1 is impaired in sexual development and incapable of producing perithecia within 7 days (Figure 3). The Δsci1 strain arrests in the sexual developmental stage of protoperithecia, has a reduced growth rate and is unable to undergo hyphal fusions [14]. These defects were not observed in the Δpom33 strain (Figure 3).

### 3.4. The Mutant Δpom33 Exhibits no Sensitivity against a Series of Stress Conditions

**Figure 4:**
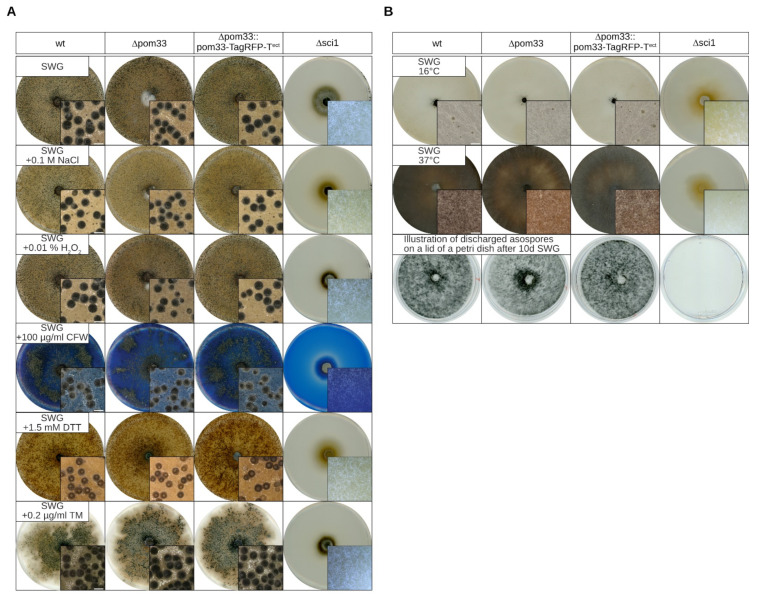
Sexual development in the wt, Δpom33, the complementation strain and Δsci1 on different stress media. (**A**) The wt, deletion mutant (Δpom33) and the complementation strain (Δpom33::pom33-TagRFP-T^ect^) as well as the Δsci1 strain were grown in presence of different stress conditions, such as osmotic- (0.1 M NaCl), oxidative- (0.01% H_2_O_2_), cell wall- (100 µg/mL CFW), or ER stress (1.5 mM DTT, 0.2 µg/mL TM) by adding the different components to SWG medium. (**B**) Strains from (**A**) were grown on SWG media at 16 °C and 37 °C to perform temperature stress. After 10 days the lids of petri dishes with discharged ascospores were documented. Here, the spores were already germinated. Pictures of the agar plates and microscopic images of perithecia as shown in the small boxes on the bottom right sides were taken after 8 days. Scale bar of microscopic images: 0.5 mm.

Since there were no obvious developmental defects of Δpom33 visible in comparison to the wt and the complemented strain, we tested sexual development under different stress conditions. Here, sexual development of all three strains including the *sci1* deletion mutant, Δsci1, was investigated after 8 days on medium containing 0.1 M NaCl, mimicking osmotic stress, or under oxidative-stress conditions by supplementing the media with 0.01% H_2_O_2_. Moreover, growth under cell wall stress (100 µg/mL CFW) or under ER-stress conditions (1.5 mM DTT, 0.25 µg/mL TM) as well as temperature sensitivity (16 °C, 37 °C) was analyzed. All strains except Δsci1 were able to form perithecia under osmotic-, oxidative-, cell wall- and ER-stress conditions displaying normal sexual development (Figure 4A). Furthermore, all strains were sensitive to cold and heat stress conditions and unable to produce mature fruiting-bodies (Figure 4B). In addition, no obvious difference in the number of discharged ascospores after 10 days could be observed except for Δsci1 (Figure 4B). These results showed that sexual development of *S. macrospora* is independent of POM33 function.

### 3.5. Proteins of the ER Are Putative Interaction Partners of POM33

**Figure 5:**
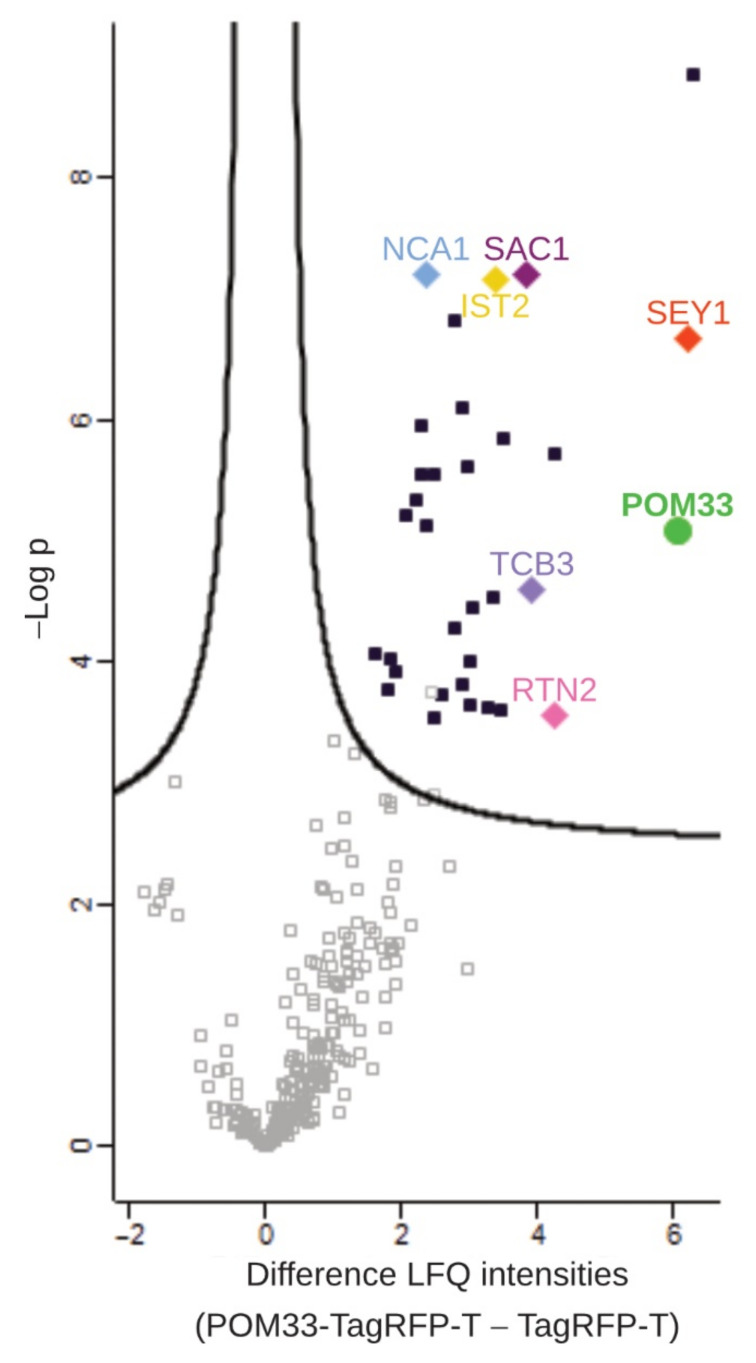
Volcano plot of putative interacting proteins from POM33-TagRFP-T pulldowns. For the pulldowns, in the first experiment three biological replicates of Δpom33::pom33-TagRFP-T^ect^ and wt::TagRFP-T^ect^ as control and for the second experiment three biological plus one technical replicate each of the sample and control strain were grown in liquid BMM media over 3 days at 27 °C under continuous light. Protein extracts of the strains were subjected to TagRFP-T-trap pulldown, trypsin digested and resulting peptides were analyzed by LC/MS. The result of two independent experiments is shown. In the volcano plot, the difference of LFQ intensities of POM33-TagRFPT in comparison to free TagRFP-T is plotted on the x-axis versus -Log *p*-values on the y-axis using a false discovery rate of 0.01 and an S0 of 0.1. The cutoff curve indicates proteins that were significantly enriched with POM33-TagRFP-T. Missing values were replaced four times with imputed values to get reliable interaction candidates. Proteins that were significant in all four repetitions are visible in the upper right part and are marked by dark blue squares (see also Table S4 in Supplementary Materials). They include NCA1 (light blue diamond), RTN2 (pink diamond), SEY1 (red diamond), TCB3 (purple diamond), IST2 (yellow diamond) and SAC1 (magenta diamond) as putative POM33 (neon green circle) interaction partners.

To analyze putative interaction partners of POM33-TagRFP-T, total protein extracts from Δpom33::pom33-TagRFP-T^ect^ strains and the control strain wt::TagRFP-T^ect^ were enriched in TagRFP-T pulldown experiments, trypsin digested and resulting peptides were analyzed by LC/MS. Data were evaluated in a semiquantitative method with label-free quantification (LFQ) based on signal intensities using the MaxQuant and Perseus programs [52,54]. Putative interaction candidates were illustrated in a volcano plot, where the difference in LFQ intensities versus the significance is graphed using a false discovery rate (FDR) of 0.01 and an S0 of 0.1 (Figure 5). To get reliable interaction partners, missing values were replaced four times with imputed values and proteins that were significant in all four repetitions are presented in Table S4 in Supplementary Materials.

Homologous proteins in *S. cerevisiae* were identified using the *S. cerevisiae* Genome Database (SGD) (https://www.yeastgenome.org/blast-sgd; accessed on 12 April 2021) [56] and for proteins in *N. crassa* or *H. sapiens*, the UniProtKB BLAST tool (https://www.uniprot.org/blast/ accessed on 12 April 2021) [57] for fungi and *H. sapiens* was used. Nearly all proteins identified as putative POM33 interaction partners are predicted to locate at the ER-membrane where they have functions in metabolic processes, cation transport or cellular trafficking (Table S4 in Supplementary Materials). Among the best hits, proteins having functions in ER-shaping and -organization were identified, namely SEY1 (SMAC_08906), a reticulon-like protein (SMAC_00989), a putative tricalbin (SMAC_02980) and TMEM16-like protein (SMAC_00943). Interestingly, the ER-marker and calcium-transporting ATPase SmNCA1 (SMAC_04583) was also ascertained as putative interaction candidate of POM33. Besides, proteins involved in lipid metabolism, like a cyclopropane-fatty-acyl-phospholipid synthase (SMAC_02133), or the putative phosphatidyl inositol 4-phosphatase SAC1 (SMAC_01583) were determined. Moreover, a fatty acid desaturase (SMAC_03650), an acyl-CoA desaturase 1 (SMAC_01733) and a very-long-chain 3-oxoacyl-CoA reductase (SMAC_01146) which play roles in fatty acid biosynthesis were identified. Additionally, several proteins participating in sterol biosynthesis were among the 33 high confidence interactors, like a sterol 24-C methyltransferase (SMAC_01214), cytochrome P450 51 (SMAC_05737), a squalene monooxygenase (SMAC_00934), a C-3 sterol dehydrogenase/C-4 decarboxylase (SMAC_04350), a C-4 methylsterol oxidase (SMAC_00952), a squalene synthase (SMAC_07413) and cytochrome P450 4A5 (SMAC_05894). Furthermore, proteins that are important for protein glycosylation were detected, like dolichyl-diphosphooligosaccharide-protein glycosyltransferase subunit 1 (SMAC_05819), a dolichyl-phosphate-mannose-protein mannosyltransferase (SMAC_02375), a dolichyl-diphosphooligosaccharide-protein glycosyltransferase subunit WBP1 (SMAC_01348) and two other dolichyl-phosphate-mannose-protein mannosyltransferases (SMAC_06805, SMAC_08483). Proteins that translocate proteins to the ER were also found, like signal recognition particle (SRP) receptor subunit beta (SMAC_09603), transport protein SEC61 subunit alpha (SMAC_05120) and a translocation complex component (putative SEC63) (SMAC_02255). The putative interaction partners of POM33 validated the result of the localization study. To examine whether the ER-morphology changed due to deletion of *Smpom33*, we transformed the Δpom33 mutant with the *Tag-RFP-T* tagged ER-marker gene *Smnca1*. However, we did not observe any difference in ER-morphology when comparing the localization of NCA1-TagRFP-T in Δpom33, Δsci1 and the wt (Figure S7 in Supplementary Materials).

As these results revealed, no SmSTRIPAK-component was found to be significantly enriched in the POM33 pulldown experiments. In addition, fluorescence microscopic co-localization studies using POM33-TagRFP-T and SCI1-EGFP indicated no obvious co-localization of the two proteins (Figure S8 in Supplementary Materials). SCI1 and the striatin protein PRO11 were shown to localize around the nucleus and at cytoplasmic structures [14]. Therefore, we analyzed their localization in the Δpom33 deletion background together with red-labeled histone 2B using fluorescence microscopy (Figure S9 in Supplementary Materials). The expression of the proteins was verified by Western blot analysis (Figure S9 in Supplementary Materials). Due to the fact that SCI1-EGFP and PRO11-EGFP signals revealed no change in localization in comparison to the wt, we conclude that localization of the two SmSTRIPAK-components SCI1 and PRO11 did not depend on SmPOM33.

## 4. Discussion

In *S. macrospora*, the striatin homolog PRO11, SCI1 and other core components of the SmSTRIPAK have been to some extend localized around the nucleus [9,14]. A protein similar to the nucleoporin POM33 had previously been identified as a putative interactor of the *S. macrospora* SmSTRIPAK component SCI1 [14], leading to the hypothesis that it might be a putative anchor of the SmSTRIPAK to the nucleus. In this study, we determined the subcellular localization of SmPOM33 and identified its putative interaction candidates via pulldown experiments coupled to LC/MS analysis. Furthermore, we successfully established the Δku80 deletion strain as a useful tool for the generation of *S. macrospora* knockout strains.

Fluorescence microscopic investigations revealed SmPOM33 localization around the nucleus, at the NE and the ER using labeled histones or NE/ER marker proteins (Figure 2A–C). The homolog of the *N. crassa* SERCA-type Ca^2+^-ATPase NCA-1, SmNCA1, was used as marker protein for the ER and the NE (Figure 2B). This calcium pump was chosen as marker because fluorescence microscopy of *N. crassa* NCA1-GFP showed co-localization with GRP-78, a protein that facilitates protein folding in the ER [66], and DPM, a dolichol-phosphate mannosyltransferase located in the ER [67,68]. Since SmNCA1 and SmPOM33 displayed co-localization, whereas the TM Nup SmPOM152 and SmPOM33 could only partially be co-localized, SmPOM33 might rather be a NE/ER protein than an NPC component (Figure 2D). In fact, *S. cerevisiae* ScPom33p was shown to localize to the inner (INM) and outer (ONM) nuclear membrane of the NE with minor fractions at the ER, whereas its paralog ScPer33p was shown to be associated with NPCs but could mainly be localized at the ER and the NE [30,69]. More precisely, ScPom33p behaves as a dynamic TM Nup, because it exchanges between the NPC and the ER using ScNdc1p and a subunit of the translocon localizing to the cortical and perinuclear ER, ScSec61p, as controls in photobleaching experiments [30]. Moreover, the authors illustrated that ScPom33p contributes to NPC assembly, distribution and stabilization. In contrast, the mammalian ortholog of ScPom33p/ScPer33p, HsTMEM33, was not demonstrated to localize at the NPC but together with Calnexin and an ER-Tracker its subcellular location was determined at the ER with enrichment at the NE [30,69,70]. Similarly, in *S. pombe* Tts1p localizes to the tubular ER network and the NE with partially enrichment at NPCs [31,71].

Our results showed that deletion of *Smpom33* has no defect in sexual development under normal or stress conditions (Figure 3 and Figure 4). Contrary to this, deletion of *sci1* displays a severe defect in sexual development and growth demonstrating that SmPOM33 likely acts independently of the SmSTRIPAK. Similarly, *S. cerevisiae* Δpom33 is viable with no growth defect at any temperature but shows a clustering-phenotype in which NPC-distribution along the NE is altered [30]. This clustering-phenotype could also be observed in yeast Δrtn1 and Δypo1 mutants that are impaired in NPC biogenesis [72,73]. Moreover, no detectable phenotype was visible upon HsTMEM33-depletion [30]. These observations suggest a redundancy between transmembrane Nups. However, HsTMEM33 protein expression was increased by ER stress induced by thapsigargin and tunicamycin implementing a role in the unfolded protein response (UPR) pathway [70]. Here, we could not observe any phenotype of Δpom33 under ER-stress conditions after application of DTT or tunicamycin in comparison to the wt (Figure 4).

**Figure 6:**
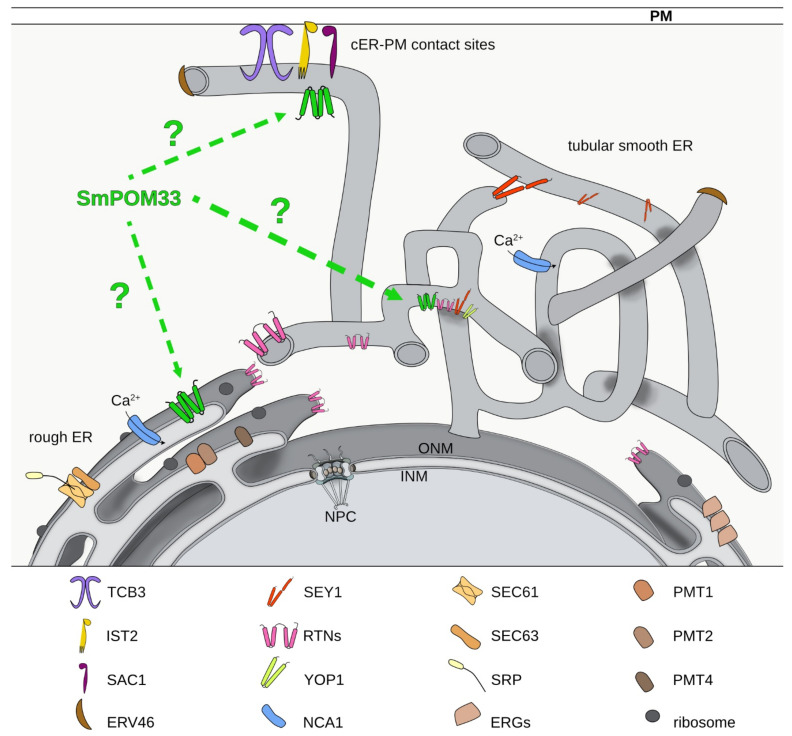
Schematic illustration of putative SmPOM33 localization and its potential interaction partners. Shown are the putative localization of SmPOM33 and the discussed proteins identified via pulldown experiments coupled to LC/MS analysis (Table S4 in Supplementary Materials). Putative interactions partners of SmPOM33 are located at the ER-membrane of the rough or tubular smooth ER. NPC: nuclear pore complex, INM: inner nuclear membrane, ONM: outer nuclear membrane, PM: plasma membrane, cER: cortical ER.

Our pulldown experiments coupled to LC/MS analysis revealed mainly ER-membrane proteins as putative interaction partners of SmPOM33 (Figure 6 and Table S4 in Supplementary Materials).

With the chosen thresholds, 33 candidates were found whereby the SmPOM152 was filtered out as it was only identified in two of the seven SmPOM33-TagRFP-T samples and therefore not covered as being significant. This result matched the observation that SmPOM33 only partially co-localized with POM152 (Figure 2D). On the other hand, a reticulon-like protein homologous to *S. cerevisiae* Rtn2p and the ER-shaping and organization component SEY1 was identified with high significance.

Formation and maintenance of highly curved ER tubules is facilitated by ER-membrane proteins like members of the reticulon (RTN) family and the DP1/Yop1p family. RTN and DP1 protein families are involved in ER tubule shaping and stabilization by inserting their wedge-shaped hairpin C-terminal region (~200 amino acids) into the outer leaflet of the lipid bilayer to deform the ER membrane [74,75,76,77]. Noteworthy, the homolog of the yeast Yop1p, SMAC_06633, was also identified in our pulldown analysis with SmPOM33 but was not enriched with high significance in the tested samples (Figure 6). In yeast and mammals, RTNs are absent from ER sheets and peripheral ER and restricted to the tubular ER network [75]. In addition, several studies suggested that RTNs and DP1/Yop1p are involved in NE assembly and *de novo* nuclear-pore formation and stability due to their membrane-shaping properties [73,78,79]. The mammalian reticulon Rtn4a/NogoA is required for the generation and maintenance of tubular ER in vitro [75]. Affinity chromatography revealed that HsTMEM33 co-localizes with Rtn4C at ER sheets and partially at ER tubules and suppresses the membrane-shaping activity of reticulons, thus regulating the tubular ER structure [32]. The *S. pombe* Tts1p co-localizes with Rtn1p and Yop1p at curved ER membranes and at the NE and plays a role in remodeling the NE during mitosis by modulating NPC distribution [31,71]. To sustain the highly curved ER domains, Ttsp1 functionally interacts with the ER-shaping proteins Rtn1p and Yop1p via its C-terminal α-helix [31,71]. For SmPOM33 also, C-terminal α-helices were predicted and an RTN2 homolog (SMAC_00989) was identified in the pulldowns. These findings hint to a possible interaction between the SmPOM33 helices and RTN2. The cooperation between TMDs and C-terminal helices in maintaining ER morphology has been reported for the dynamin-like GTPase atlastin/Sey1p [80,81,82]. In the study presented here, the homolog of Sey1p, SMAC_08906, was identified as a putative interactor of SmPOM33 (Table S4 in Supplementary Materials and Figure 5). Studies from [80] showed that mammalian atlastins localize to tubular ER and interact with tubule-shaping proteins. The atlastins functional ortholog in *S. cerevisiae*, Sey1p, mediates the fusion of two ER tubules at three-way junctions [80,83].

Additionally, to these ER-shaping candidates, SmNCA1 (SMAC_04583) occurred among the best hits (Figure 5 and Figure 6). This result hint to a possible interaction of the Ca^2+^-ATPase NCA1 with SmPOM33, which matched with their co-localization in fluorescence microscopy (Figure 2B). In *S. cerevisiae*, the P-type Ca^2+^-ATPase Pmr1p, transports calcium and manganese into the Golgi [84,85,86].

Furthermore, *S. macrospora* homologs of *S. cerevisiae* tricalbin Tcb3p (SMAC_02980), the phosphatidylinositol phosphate phosphatase Sac1p (SMAC_01583) and the cER protein Ist2p (SMAC_000943) were identified (Figure 5 and Figure 6). In yeast, together tricalbins and Ist2p are involved in tethering cER to the plasma membrane (PM) at cER-PM contact sites. They control the access of the phosphatydil-inositol-4-phosphatase Sac1p to its substrate phosphatidylinositol-4-phosphate (PI4P) thus regulating PI4P levels [87]. Thereby, contributing to cellular lipid fluxes, shaping of the cER and PM integrity [88,89]. Interestingly, a recent global phosphoproteom study of SmSTRIPAK mutants revealed that the tricalbin homolog SMAC_02980 is differential phosphorylated at T1203 in STRIPAK mutants making this SmPOM33 interactor a putative STRIPAK target [90]. In *S. cerevisiae*, the SmPOM33 homolog Per33p showed a positive genetic interaction with Tcb3p suggesting a regulatory connection between both proteins [91].

Additionally, putative homologs of SEC61 (SMAC_05120) and SEC63 (SMAC_02255) together with the SRP receptor beta subunit (SMAC_09603) were enriched in the SmPOM33 pulldown (Table S4 in Supplementary Materials). The Sec61 complex mediates SRP-dependent protein import into the ER and by interaction with the Sec63 complex is required for SRP-independent protein import [92,93]. Moreover, three putative protein O-mannosyltransferases homologous to yeast PMT1 (SMAC_06805), PMT2 (SMAC_02375), and PMT4 (SMAC_08483) were found to be enriched in the LC/MS analysis of SmPOM33 pulldowns. In *S. cerevisiae,* Pmts are located in the ER membrane where they function in protein O-glycosylation [94,95,96]. Besides these ER membrane proteins, a homolog of the *S. cerevisiae* COPII-coated vesicle protein ERV46 (SAMC_04901) was identified. In fact, specialized regions of the smooth tubular ER, called ER exit sites (ERES), play a unique role in the assembly of COPII-coated vesicles which mediate trafficking from ER to the Golgi [97]. Besides, several *S. macrospora* homologs of the ergosterol-biosynthesis pathway were also enriched in the pulldown experiments, namely ERG6 (SMAC_01214), ERG11 (SMAC_05737 or SMAC_05894), ERG1 (SMAC_00934), ERG26 (SMAC_04350), ERG25 (SMAC_00952) and ERG9 (SMAC_07413). Of interest, in *N. crassa* the knockout of *erg2* or *erg1* leads to cell fusion defects of hyphae [98,99]. This phenotype of dysfunctional hyphal fusion was also described for mutants of SmSTRIPAK components like in Δsci1 [14].

The results of the pulldown using SmPOM33 as bait revealed no SmSTRIPAK-component as putative interaction partner. Therefore, it seems that SmPOM33 is not anchoring the SmSTRIPAK to the nucleus. This suggestion is highlighted by the fact, that using PRO11 and SmMOB3 as bait, the nucleoporin NIC96 of the NPC was identified and might be the candidate responsible for nucleus-anchoring of the SmSTRIPAK [14]. Furthermore, SCI1 and PRO11 were co-localized with the nucleoporin SmPOM152 [14] that only partially co-localized with SmPOM33. Interestingly, SmSEY1 (SMAC_08906) was significantly enriched in PRO11 pulldowns [14].

The results of our fluorescence microscopic localization studies and pulldown experiments coupled to LC/MS analysis indicate that similar to the human homolog HsTMEM33, SmPOM33 is rather an ER/NE protein than a component of the NPC. HsTMEM33 is absent from the NPC and abundant at the ER and NE [30,69]. Targeting of ScPom33p to the NPC is facilitated by the nuclear import factor Kap123 that directly interacts with the amphipathic α-helices within the C-terminal domain of ScPom33p [69]. Accordingly, proper NPC localization of ScPom33p is dependent on its lipid binding ability and Kap123 binding. Interestingly, the CTD of ScPom33p is not very conserved in SmPOM33, revealing that SmPOM33 possesses no binding site for the direct interaction with nuclear import factors. This fact is underlined by the results of the LC/MS analysis, where mainly ER-membrane proteins were identified and no component of NPCs or any nuclear import factor like Kap123.

## 5. Conclusions

In conclusion, the pulldown and LC/MS analysis of SmPOM33 revealed potential interactions partners that are localized at the ER-membrane in the nuclear envelope or at cER-PM contact sites possessing different cellular functions (Figure 6). Therefore, SmPOM33 is rather an ER-protein than a component of the NPC. To confirm this assumption, further future studies are required to verify direct physical or genetic interaction with the identified candidates and the impact of the STRIPAK complex on ER morphology and organization. These include the identification of the conditions under which SmPOM33 is functional, the exact localization of SmPOM33 by super-resolution microscopy or immune EM.

## Figures and Tables

**Table 1:** Strains used and generated in this study.

Strain	Genotype	References
*Escherichia coli*		
MACH1	Δ*recA1398*, *endA1*, *tonA*, *Φ80*Δ*lacM15*, Δ*lacX74*, *hsdR*, (rK-mK+)	Invitrogen
*Saccharomyces cerevisiae*		
PJ69-4A	*MATa*, *trp1-901*, *leu2-3*, *112*, *ura3-52*, *his3-200*, *gal4*Δ, *gal80*Δ, *LYS2::GAL1-HIS3*, *GAL2-ADE2*, *met2::GAL7-lacZ*	[35]
*Sordaria macrospora*		
DSM997	wild type (wt)	DSMZ
S23442	mutation in *fus1-1* gene, brownish ascospores, fertile	[41]
Δku70	Δku70::nat^R^, fertile	[42]
Δsci1	Δsci1::hyg^R^, ssi, sterile	[14]
Δsci1::nca1-TagRFP-T^ect^	ectopic integration of p5′nca1-TagRFP-T _nat into Δsci1; *hyg^R^*, *nat^R^* pt, sterile, *Pnca1::nca1::TagRFP-T::TtrpC*	Reschka and Pöggeler (unpublished)
wt::egfp^ect^	ectopic integration of p1783-1 into DSM997; *hyg^R^*, ssi, fertile, *Pgpd::egfp::TtrpC*	[43]
wt::TagRFP-T^ect^	ectopic integration of pTagRFP-T_nat into DSM997; *nat^R^*, ssi, fertile, *Pccg1::TagRFP-T::TtrpC*	[44]
wt::GH2A^ect^	ectopic integration of pGH2A into DSM997; *hyg^R^*, pt, fertile, *Pgpd::hh2a::egfp::TtrpC*	Reschka and Pöggeler (unpublished)
fus::RH2B^ect^	ectopic integration of pRH2B in S23442; *hyg^R^*, pt, fertile, *Pgpd::hh2b::tdTomato::TtrpC*	Reschka and Pöggeler (unpublished)
wt::nca1-TagRFP-T^ect^	ectopic integration of p5′nca1-TagRFP-T_hyg into DSM997; *hyg^R^*, pt, fertile, *Pnca1::nca1::TagRFP-T::TtrpC*	Werner and Pöggeler (unpublished)
Δku80	Δku80::hyg^R^, ssi, fertile	This study
Δpom33	Δpom33::nat^R^, ssi, fertile	This study
wt::pom33-TagRFP-T^ect^	ectopic integration of p5′pom33-TagRFP-T_nat into DSM997; *nat^R^*, ssi, fertile, *Ppom33::pom33:: TagRFP-T::TtrpC*	This study
wt::pom33-TagRFP-T^ect^	ectopic integration of p5′pom33-TagRFP-T_hyg into DSM997; *hyg^R^*, pt, fertile, *Ppom33::pom33:: TagRFP-T::TtrpC*	This study
wt::pom33-egfp^ect^	ectopic integration of p5′pom33-egfp into DSM997; *nat^R^*, ssi, fertile, *Ppom33::pom33::egfpt::TtrpC*	This study
wt::pom33-TagRFP-T^ect^ + pom152-egfp^ect^	ectopic integration of p5′pom33-TagRFP-T_hyg and pSmPOM152GFP into DSM997; *hyg^R^*, *nat^R^*, pt, fertile, *Ppom33::pom33:: TagRFP-T::TtrpC; Pccg1::pom152::egfp::TtrpC*	This study
wt::pom33-TagRFP-T^ect^ + sci1-egfp^ect^	ectopic integration of p5′pom33-TagRFP-T_hyg and p5′sci1gfp_nat into DSM997; *hyg^R^*, *nat^R^*, pt, fertile, *Ppom33::pom33:: TagRFP-T::TtrpC; Psci1::sci1::egfp::TtrpC*	This study
Δpom33::pom33-TagRFP-T^ect^	ectopic integration of p5′pom33-TagRFP-T_hyg into Δpom33; *hyg^R^, nat^R^* ssi, fertile, *Ppom33::pom33:: TagRFP-T::TtrpC*	This study
Δpom33::RH2B^ect^	ectopic integration of pRH2B into Δpom33; *hyg^R^, nat^R^* pt, fertile, *Pgpd::hh2b::tdTomato::TtrpC*	This study
Δpom33:: nca1-TagRFP-T^ect^	ectopic integration of p5′nca1-TagRFP-T_hyg into Δpom33; *hyg^R^*, *nat^R^* pt, fertile, *Pnca1::nca1::TagRFP-T::TtrpC*	This study
Δpom33::sci1-egfp^ect^	ectopic integration of p5′sci1-egfp_hyg into Δpom33; *hyg^R^, nat^R^* pt, fertile, *Psci1::sci1::egfp::TtrpC*	This study
Δpom33::pro11-egfp^ect^	ectopic integration of pPRO11-GFP_hyg into Δpom33; *hyg^R^, nat^R^* pt, fertile, *Pccg1::HA::pro11::egfp::TtrpC*	This study

*nat^R^*: nourseothricin resistant, *hyg^R^*: hygromycin resistant, *Pccg1*: promotor of the *clock controlled gene 1* of *Neurospora crassa*, *Pgpd*: promotor of the glyceraldehyde-3-phosphate dehydrogenase gene of *Aspergillus nidulans*, *TtrpC*: terminator of the anthranilate synthase gene of *A. nidulans,* ssi: single-spore isolate, pt: primary transformant, *egfp*: gene for green fluorescent protein enhanced green fluorescent protein (eGFP) of *Aequoria victoria*, *TagRFP-T*: gene for red fluorescent protein TagRFP-T of *Entacmaea quadricolor,* *tdTomato*: gene for red fluorescence protein tdTomato from *Discosoma* species. *P*: promoter, *T*: terminator.

## Data Availability

The mass spectrometry proteomics data have been deposited to the ProteomeXchange Consortium via the PRIDE [100] partner repository with the dataset identifier PXD026253.

## Supplementary Materials

The following are available online at https://www.mdpi.com/article/10.3390/jof7090682/s1, Table S1: list of plasmids, Table S2: primers used in this study, Table S3: Perseus 1.6.0.7 workflow, Figure S1: transmembrane nucleoporins and domain organization of POM33 orthologs and paralogues in human and fungi, Figure S2: localization of free TagRFP-T and EGFP, Figure S3: fluorescence microscopy of POM33-TagRFP-T/-EGFP and expression controls via Western blot analysis, Figure S4: verification of *Smku80* deletion by PCR and Southern blot analyses, Figure S5: sexual development and vegetative growth rates in the Δku80 and wt strain, Figure S6: verification of *Sm**pom33* deletion by PCR and Southern blot analyses, Figure S7: localization of the ER-marker NCA1-TagRFP-T in the *S. macrospora* wt, Δpom33 and Δsci1 strain, Figure S8: co-localization of POM33 and SCI1 in *S. macrospora* wt, Figure S9: localization of SCI1 and PRO11 in Δpom33 and Western blot analysis.
