# Supplementary material for: Analysis of the Putative Nucleoporin POM33 in the Filamentous Fungus Sordaria macrospora
Source: J Fungi (Basel). 2021 Aug 24;7(9):682. doi: 10.3390/jof7090682 (PMC8468769; doi:10.3390/jof7090682)
Supplement: Supplementary file 1 [file jof-07-00682-s001.zip › PRIDE Protocol.pdf]

Summary

Title

Analysis of the Putative Nucleoporin POM33 in the Filamentous Fungus *Sordaria macrospora*

Description

The POM33 protein of *Sordaria macrospora* is a nucleoporin considered of being an ER-protein that may function in ER-shaping and organization. In this study, we performed TagRFP-T pulldown experiments in *S. macrospora* strains expressing either POM33-TagRFP-T or the free TagRFP-T as control to identify putative POM33 interaction partners. Since with the SmSTRIPAK-component SCI1, POM33 was found, we expected with this approach to *vice versa* pull down SCI1. The results of the LC/MS analysis revealed mainly proteins that are located at the ER-membrane where they have functions in fatty acid biosynthesis, sterol biosynthesis, lipid metabolism or ER-shaping and maintaining its organization. These results gave us an overall picture about putative interactions of POM33 to specify its localization and function in *S. macrospora* hyphae.

Sample Processing Protocol

Three biological and one technical replicate of the *S. macrospora* strains wt::TagRFP-T^{ect} (control) and Δpom33::pom33-TagRFP-T^{ect} (sample) were used in two independent experiments. Strains were cultivated in liquid biomalt maize medium (BMM) for 3 d at 27 °C. Harvested mycelium was ground in liquid nitrogen and after adding 520 µl lysis buffer (10 mM Tris-HCl pH 7.5, 150 mM NaCl, 0.5 mM EDTA pH 8.0, 1 mM PMSF, 2 mM DTT, 1x protease inhibitor cocktail IV (1tbl/50 ml, 04693132001, Mannheim, Germany), 1x PhosSTOP™ (1tbl/10 ml, Roche, 04906837001, Mannheim, Germany)) / g mycelium powder and ~200 µl glass beads (Ø 0.25-0.5 mm, Roth GmbH A553.1), cells were vortexed for 2 min. Following centrifugation at 10.000 g for 20 min. at 4 °C the lysates were centrifuged again at 10.000 g for 10 min. at 4 °C. Wt-lysate was used to dilute the control 1:1.

For the pulldown, 1 ml of the lysate was incubated with 2 µl of primary TagRFP-T (rabbit) antibody for 2 h at 4 °C on a rotation wheel. Lysate-antibody solution was added to 50 µl Dynabeads™ Protein G (Invitrogen, 10003D) and rotated for 1 h at 4 °C. The magnet was applied, supernatant removed and beads washed twice with dilution buffer (lysis buffer without NP-40 and DTT). To separate beads from the antibody-protein complex, 50 µl of 4x NuPAGE® LDS-SB (Invitrogen, NP0007) was added to get a 1x dilution plus 5 µl 1 M DTT and heated for 10 min. at 70 °C. 25 µl of the control and samples were loaded on a 12 % SDS gel and sample lanes were separated by lanes loaded with 5 µl of the Nippon Genetics Co. Europe blue star pre-stained protein marker (NIPPON Genetics Europe, MWP03, Düren, Germany). Subsequently, the gel was shaken for 30 min. in fixing solution (10 % acetic acid and 40 % ethanol) and washed with H_2O for 10 min.

Sample and control lanes were cut into 4 small pieces and subjected to tryptic in-gel digestion according to Shevchenko et al., 1996. Purification of peptides was achieved using C18 stage tips according to Rappsilber et al., (2003). The peptide solution was dried completely in the SpeedVac concentrator (Eppendorf concentrator 5301) and the pellet was resolved in 20 µl of sample buffer (2 % acetonitrile, 0.1 % formic acid) for LC/MS analyses.

For peptide separation, 2 µl of each sample were subjected to reverse phase liquid chromatography using an RSLCnano Ultimate 3000 system (Thermo Fisher Scientific). Peptides were loaded on an Acclaim PepMap 100 pre-column (100 µm x 2 cm, C18, 5 µm, 100 Å; Thermo Fisher Scientific) with 0.07 % trifluoroacetic acid at a flow rate of 20 µl/min

for 3 min. To separate peptides analytically, an Acclaim PepMap RSLC column (75 µm x 50 cm, C18, 2 µm, 100 Å; Thermo Fisher Scientific) with a flow rate of 300 nl/min. was used. The solvent composition was gradually changed within 94 min from 96 % solvent A (0.1 % formic acid) and 4 % solvent B (80 % acetonitrile, 0.1 % formic acid) to 10 % solvent B within 2 minutes, to 30 % solvent B within the next 58 min, to 45 % solvent B within the following 22 min, and to 90 % solvent B within the last 12 min of the gradient. All solvents and acids were prepared to have Optima grade for LC/MS (Thermo Fisher Scientific). Nano-electrospray (nESI) using the Nanospray Flex Ion Source (Thermo Fisher Scientific) at 1.5 kV (liquid junction) was used to on-line ionize eluting peptides, which were subsequently transferred into a Q Exactive HF mass spectrometer (Thermo Fisher Scientific). At a resolution of 30,000, full scans in a mass range of 300 to 1650 m/z were recorded followed by data-dependent top 10 HCD fragmentation at a resolution of 15,000 (dynamic exclusion enabled). For data acquisition and programming the XCalibur 4.0 software (Thermo Fisher Scientific) was used.
References: Shevchenko A, Wilm M, Vorm O, Mann M. 1996. Mass spectrometric sequencing of proteins silver-stained polyacrylamide gels. Anal. Chem. 68:850-858. Rappsilber J, Ishihama Y, Mann M. 2003. Stop and Go Extraction Tips for Matrix-Assisted Laser Desorption/Ionization, Nanoelectrospray, and LC/MS Sample Pretreatment in Proteomics. Anal. Chem. 75,3:663-670.

Data Processing Protocol

Protein identification was performed with the MaxQuant 1.6.0.16 software (Cox & Mann, 2008). The *S. macrospora*-specific peptide database Smacrospora_v03 (Blank-Landeshammer et al., 2019), including the peptide sequence of free TagRFPT, was used for database search with the Andromeda algorithm and the program's default parameters. As the digestion mode trypsin/P was used, maximum missed cleavage sites were set to two, carbamidomethylation of cysteins was considered as fixed modification, acetylation of the N-terminus and oxidation of methionines were set as variable modifications. Label free quantification (LFQ) was activated with a minimal ratio count of two. The decoy mode was revert with a false discovery rate of 0.01. Data processing and statistical analysis was performed with the Perseus 1.6.0.7 software (Tyanova et al., 2016).

References: Cox J, Mann M. 2008. MaxQuant enables high peptide identification rates, individualized p.p.b.-range mass accuracies and proteome-wide protein quantification. Nat. Biotechnol. 26:1367-1372. Tyanova S, Temu T, Sinitcyn P, Carlson A, Y Hein M, Geiger T, Mann M, Cox J. 2016. The Perseus computational platform for comprehensive analysis of (prote)omics data. Nature Methods. 13:731-740.
