# Supplementary material for: Analysis of the Putative Nucleoporin POM33 in the Filamentous Fungus Sordaria macrospora
Source: J Fungi (Basel). 2021 Aug 24;7(9):682. doi: 10.3390/jof7090682 (PMC8468769; doi:10.3390/jof7090682)
Supplement: Supplementary file 1 [file jof-07-00682-s001.zip › Supplement.pdf]

Supplementary material

Table S1: List of plasmids

Plasmid	Feature	Reference
pRS426	*amp*^{R}, *ura3*	[1]
pCB1003	*amp*^{R}, *hph*^{R}	[2]
pRS-nat	*amp*^{R}, *ura3, nat*^{R}	[3]
pRS-hyg	*amp*^{R}, *ura3, hyg*^{R}	[4]
pGG-nat1	*amp*^{R} *Bsa*I *(5)::PtrpC::nat1::Bsa*I *(6)* in pJet1.2	[5]
pDest-Amp	Destination vector for Golden Gate cloning*; bla (Bsamut),* *lacZ* gene with two internal *Bsa*I sites: *Bsa*I(4) and *Bsa*I(7)	[5]
p1783-1	*amp*^{R}, *ura3, hyg*^{R} *Pgpd::egfp::TtrpC*	[6]
pRH2B	*amp*^{R}, *ura3, hyg*^{R} *Pgpd::hh2b::tdTomato::TtrpC*	[7]
pTagRFP-T_nat	*amp*^{R}, *nat*^{R} *Pccg1::TagRFP-T::TtrpC*	[8]
p5'sci1gfp_nat	*amp*^{R}, *ura3, nat*^{R} *Psci1::sci1::egfp::TtrpC*	[9]
pPRO11-GFP_hyg	*amp*^{R}, *ura3, nat*^{R} *Pccg1::HA::pro11::egfp::TtrpC*	Herzog & Pöggeler (unpublished)
pSmPOM152GFP	*amp*^{R}, *ura3, nat*^{R} *Pccg1::pom152::egfp::TtrpC*	[9]
p5'nca1-TagRFP-T_nat	*amp*^{R}, *ura3, nat*^{R} *Pnca1::nca1::TagRFP-T::TtrpC*	Reschka (unpublished)
p5'nca1-TagRFP-T_hyg	*amp*^{R}, *ura3, hyg*^{R} *Pnca1::nca1::TagRFP-T::TtrpC*	Werner (unpublished)
pRSku80::hph	*amp*^{R,} *ura3* *5'-flanking region and 3'-flanking region of ku80 interrupted by hph*^{R} *in pRS426*	This study
p5'sci1-egfp_hyg	*amp*^{R}, *ura3, hyg*^{R} *Psci1::sci1::egfp::TtrpC*	This study
p5'pom33-egfp	*amp*^{R}, *ura3, nat*^{R} *Ppom33::pom33::egfp::TtrpC*	This study
p5'pom33-TagRFP-T_nat	*amp*^{R}, *ura3, nat*^{R} *Ppom33::pom33::TagRFP-T::TtrpC*	This study
p5'pom33-TagRFP-T_hyg	*amp*^{R}, *ura3, hyg*^{R} *Ppom33::pom33::TagRFP-T::TtrpC*	This study

pPom33-KO	*amp*^{R}*, 5'-flanking region and 3'-flanking region of pom33 interrupted by nat*^{R} *in pDest-Amp*	This study

nat^R: nourseothricin resistant, *hyg*^{R}: hygromycin resistant, *amp*^{R}: ampicillin resistance, *ura3*: orotidine-5'-phosphate decarboxylase gene of *Saccharomyces cerevisiae*, *nat:* nourseothricin acetyltransferase gene, *hph*: hygromycin B phosphotransferase gene, *Pccg1*: promotor of the *clock controlled gene 1* of *Neurospora crassa*, *Pgpd*: promotor of the glyceraldehyde-3-phosphate dehydrogenase gene of *Aspergillus nidulans*, *TtrpC*: terminator of the anthranilate synthase gene of *A. nidulans, 5':* native promotor of the respective gene, *egfp*: gene for green fluorescent protein enhanced green fluorescent protein (eGFP) of *Aequoria victoria*, *TagRFP-T*: gene for red fluorescent protein TagRFP-T of *Entacmaea quadricolor*. *P*: promoter, *T*: terminator

Table S2: Primers used in this study

Oligo name	Sequence (5' → 3')
5'sci1-f	***GTAACGCCAGGGTTTTCCCAGTCACGACG*** GCTAGCTATCAACATCATCG
pRS426GFPrev	***GCGGATAACAATTTCACACAGGAAACAGC*** TCGAGTGGAGATGTGGAGTG
Smpom33-f	***GTAACGCCAGGGTTTTCCCAGTCACGACG*** TGTTCCTACCTATGACCGTC
Smpom33-egfp-r	***GTGAACAGCTCCTCGCCCTTGCTCACCAT*** GGAGGTCTTCTTGGGGGCAG
RFP-f	ATGGTGTCTAAGGGCGAAGAG
Smpom33-RFP-r	***TTAATCAGCTCTTCGCCCTTAGACACCAT*** GGAGGTCTTCTTGGGGGC
Pom33-GG-ko-5f	***GACTGGTCTCA AGTC*** TCATGTCAATGATATGGTTGG
Pom33-GG-ko-5r	***CAGAGGTCTCA GCAG*** AACGGAAGAGCTGGGCGTTTT
Pom33-GG-ko-3f	***GTACGGTCTCG GTCA*** TAAGTTGTCGGCTATTGCCGA
Pom33-GG-ko-3r	***CTCAGGTCTCC CGTA*** GACTGCTGAGTGACACAGCCG
GG_KO_fw	TAGGGCGAATTGGGTACCG
GG_KO_rv	GGCCGCTCTAGAACTAGTG
Pom33-v2f	ATGGGGGATCAACAGCAACA
Smpom33-vORF3-f	GTCATCCTCTTCCAGCGC
Pom33-v2r	TCTTTTTAGCCTGAACAAGTTG
ku80-5f	***GTAACGCCAGGGTTTTCCCAGTCACGACG*** GGCAATGGGGGCCAGAACCA

ku80-5-hyg-r	***CAAAAAATGCTCCTTCAATATCAGTTAAC***<br><br>GAGGGAGCTGTTCGTTACAG
ku80-3-hyg-f	***GAGTAGATGCCGACCGGGAACCAGTTAAC***<br><br>TACGCGCCCATCGATGACGT
ku80-3r	***GCGGATAACAATTTCACACAGGAAACAGC***<br><br>ACTGAGGTGGCCCAAATCAG
hph-f	GTTAACTGATATTGAACGAGCATTTTTGG
hph-r	GTTAACTGGTTCCCGGTCGGCATCTACTC
ku80-ko-v5f	CTTCATACACTTGGTAGTAGC
tC1	CACCGCCTGGACGACTAAACC
h3	GTACTCGCCGATAGTGGAAAC
ku80-r	GTTCGATCCCCACATTATTAAGCAT
ku80-1r	GTTCGATCCCCACATTATTAAGCAT
ku80-1f	ATGGCGGACAAGGAAGCCATCG
ku80-ko-v5r	CTATCAGAAGCTGGCGATAAC
ku80-ko-v3f	CACGTACCTCACGGAACTCAA

Bold italics = overhangs

Table S3: Perseus 1.6.0.7 workflow

Step	Subgroup	Command	Description
1	*Load*	Generic matrix upload	Upload proteingroups.txt<br>Main: LFQ intensities<br>Numerical: MS/MS counts, Mol. Weight, score, peptides, razor + unique peptides, unique peptides, sequence coverage, intensity, number of proteins, sequence length, majority protein IDs, Protein IDs and oxidation (M) site positions. Parameters were loaded for every individual sample.
2	*Processing*	Filter rows based on categorical column	Remove matching rows with + for Only identified by site, Reverse and Potential contaminant
3		Rearrange	Remove empty columns
4		Transform	LFQ intensities = log2(x)
5	*Analysis*	Analysis	Multi scatter plot<br>Numeric Venn diagram
6	*Processing*	Categorical annotation rows	Groups: TagRFP-T control and examined POM33-TagRFP-T strains
7		Filter rows based on valid values	Min. valids: 7<br>Mode: In total<br>Values should be: Valid<br>Filter mode: Reduce matrix
8		Replace missing values from normal distribution	Width: 0.3<br>Down shift: 1.8<br>Mode: Total matrix
9	*Analysis*	Analysis	Volcano plot<br>First group (right): POM33-TagRFP-T<br>Second group (left): TagRFP-T control<br>Test: t-test<br>Side: Both<br>Number of randomizations: 250<br>Preserve grouping in randomizations: None<br>FDR: 0.01<br>S0: 0.1
10	Repeat Step 8 and 9 four times		
11	Significant interaction partners are loaded in the online program Venny2.1 [10] and a list of replicates found in all 4 repetitions of the statistical analysis as significant (Step 8 and 9) is generated		
12	*Analysis*	Analysis	Select rows with significant hits found in all four repetitions as co-enriched with the bait based on the result of step 11<br>Export selection (reduce matrix)
13	*Processing*	Imputation	Replace imputed values by NaN
14		Rearrange	Sort by column: LFQ intensity POM33-TagRFP-T_6<br>Descending: put a tick

15		Copy Matrix	Paste Matrix into Excel, see Figure S7
16		Select representative Volcano Plot	Label significant partners (found in all 4 repetitions)

A

Transmembrane Nucleoporins (TM Nups)		
S. cerevisiae	*H. sapiens*	*S. pombe*
-	Gp210	-
Pom152p	-	Pom152p
Pom34p	-	Pom34p
Ndc1p	Ndc1	Cut11p
-	Pom121	-
**Pom33p**	**TMEM33**	**Tts1p**

B

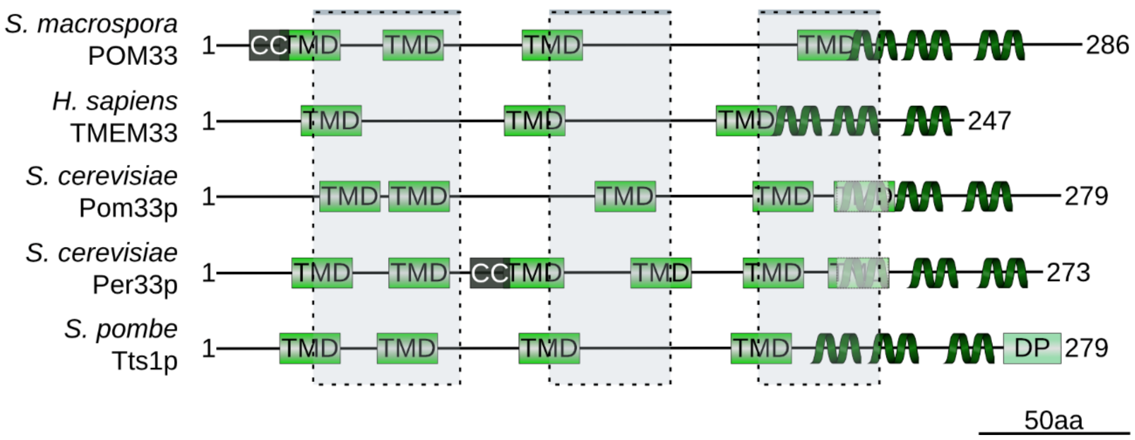

Figure S1: Transmembrane Nucleoporins and domain organization of POM33 orthologs and paralogues in human and fungi. (**A**) Transmembrane nucleoporins (TM Nups) so far known in *S. cerevisiae*, *H. sapiens* and *S. pombe*. In *S. cerevisiae* and *S. pombe* these are Pom152p/ Pom152, Pom34p/ Pom34, Ndc1p/ Cut11, and the newly identified Pom33p/ Tts1p, respectively. In *H. sapiens*, Gp210, Ndc1, Pom121 and TMEM33 belong to TM Nups. (**B**) Using the program InterProScan [11] transmembrane domains (TMD), indicated in neon green, and a disorder prediction (DP) domain for *S. pombe* Tts1p shown in light green, were predicted. The coiled-coil (CC) motifs, shown in black, were predicted using NPS@: COILED-COILS PREDICTION [12] and helices shown in dark green with the program NetSurfP – 2.0 [13]. Grey shaded boxes indicate positions of three hydrophobic regions each potentially fitting two TMDs after [14]. Accession numbers of the proteins are as following: *S. macrospora* POM33 (XP_003349881.1), *H. sapiens* TMEM33 (NP_060596.2), *S. cerevisiae* Pom33p (NP_013077.1) and its paralogue Per33p (NP_013165.1) and *S. pombe* Tts1p (NP_596818.1).

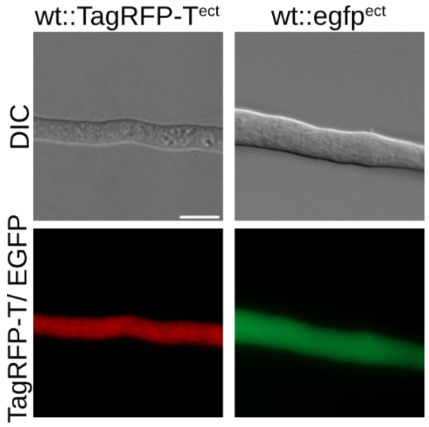

Figure S2: Localization of free TagRFP-T and EGFP. Fluorescence microscopic analysis of *S. macrospora* wt strain carrying pTagRFP-T_nat (*TagRFP-T* under control of the *Neurospora crassa ccg1* promotor [8]) or p1783-1 (*egfp* under control of the *A. nidulans gpd* promotor [6]. DIC, differential interference contrast. Scale bar = 10 µm.

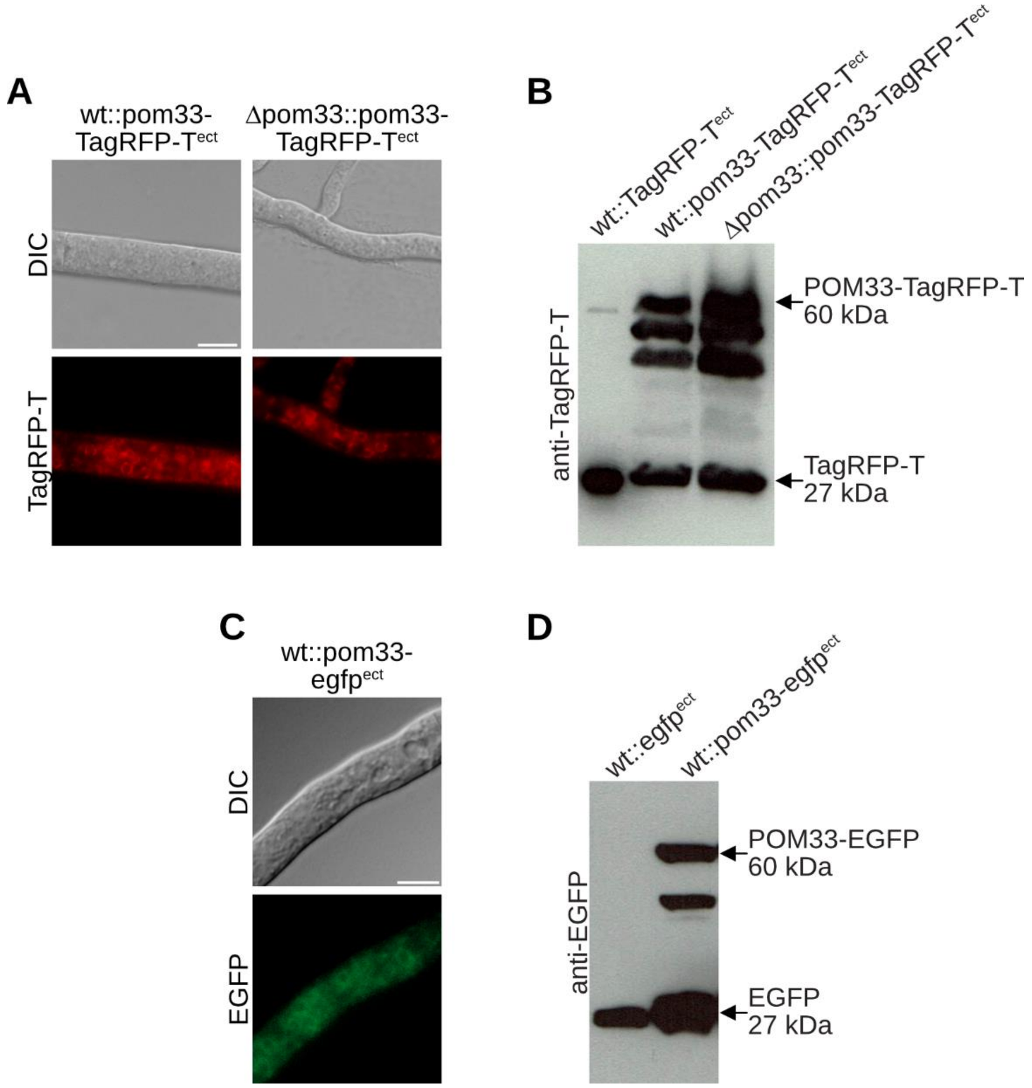

Figure S3: Fluorescence microscopy of POM33-TagRFP-T/-EGFP and expression controls via Western blot analysis. Fluorescence microscopic analysis of *S. macrospora* wt and Δpom33 expressing either POM33-TagRFP-T (**A**) or POM33-EGFP (**C**) and corresponding Western blot analyses (**B** & **D**) are shown. The strains wt::TagRFP-T^{ect} or wt::egfp^{ect} served as controls. Protein sizes are indicated. Degradation products of POM33 fusion constructs are visible in **B** & **D**. DIC, differential interference contrast. Scale bar = 10 µm.

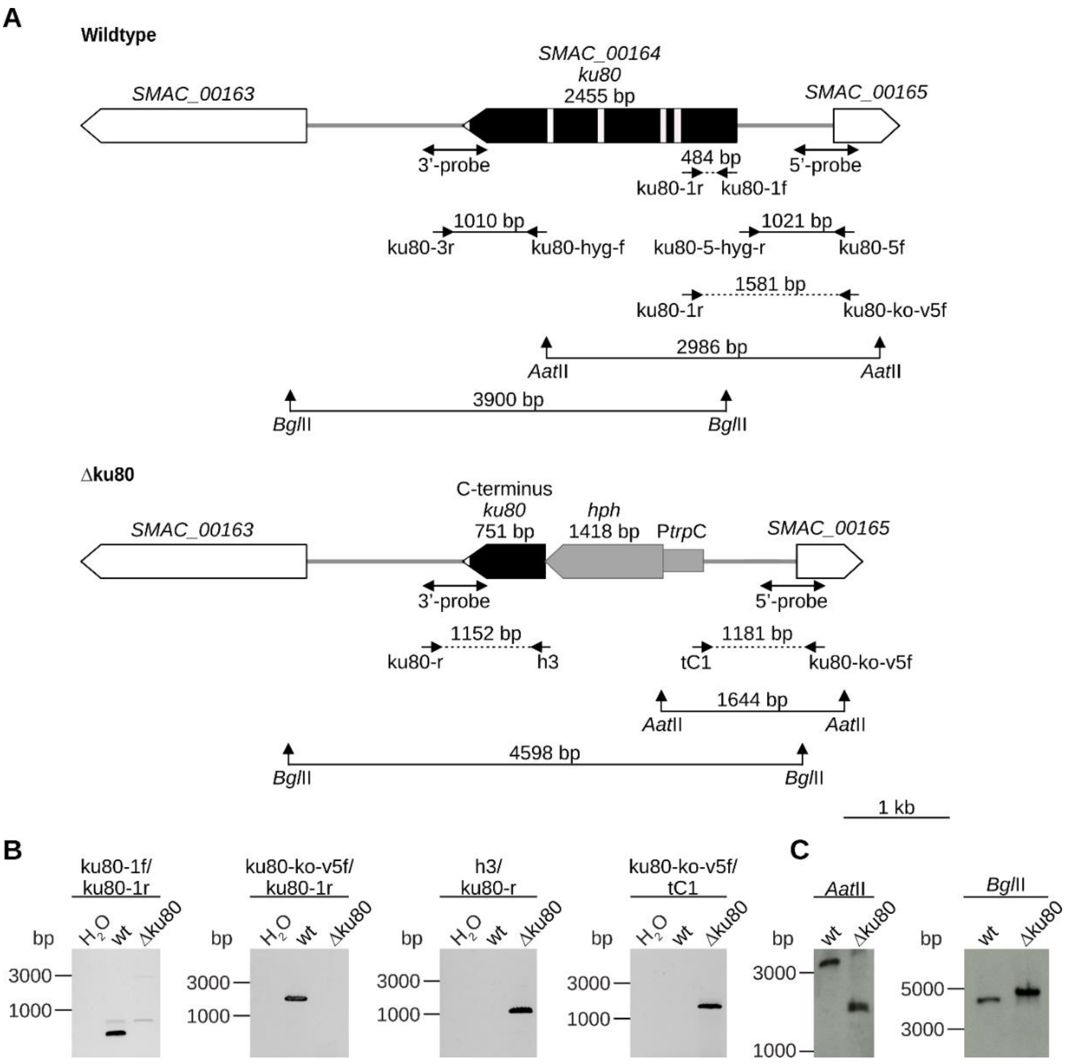

Figure S4: Verification of *Smku80* deletion by PCR and Southern blot analyses. (**A**) Schematic representation of the *Smku80* ORF *SMAC_00164* (black arrow) with 5 introns (white boxes). White arrows indicate adjacent ORFs. After homologous recombination, the *hph* cassette replaced most of the *Smku80* ORF. Primer combinations, the corresponding fragment sizes, probes for Southern hybridization and hybridization regions are indicated. (**B**) PCR verification of the homologous integration of the *hph* cassette into the *Smku80* locus. Genomic DNA was isolated from the wt and Δku80 strain and tested with given primer combinations. Water serves as negative control. (**C**) Confirmation of the *Smku80* deletion was performed by Southern blot. The probe for the 3’ flank was PCR amplified with primer pair ku80-ko-v3f x ku80-r and for the 5’ flank with primer pair ku80-ko-v5f x ku80-ko-v5r. The isolated genomic DNA was hydrolyzed with *Aat*II and *Bgl*II, respectively. Signals detected correspond to the fragment size of 3.0 kb and 3.9 kb for the wt and 1.6 and 4.6 kb for the *ku80* deletion strain.

A

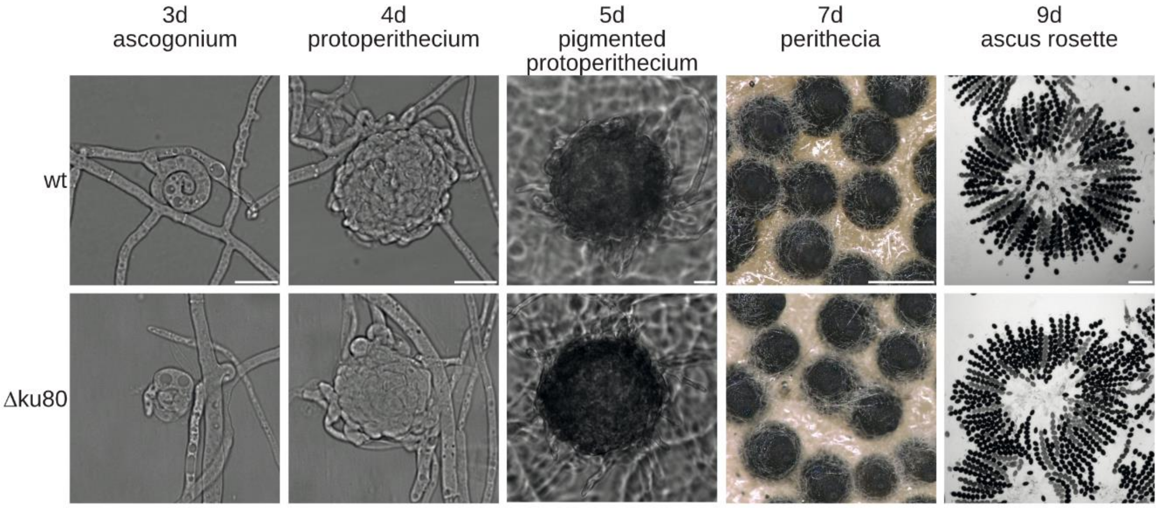

B

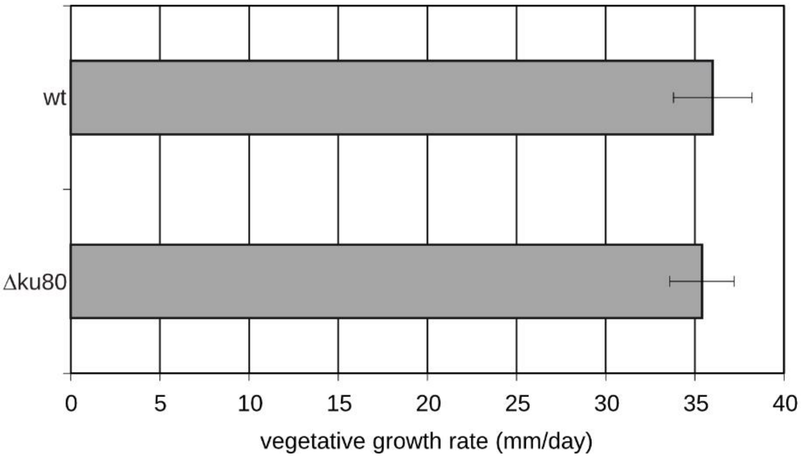

Figure S5: Sexual development and vegetative growth rates in the Δku80 and wt strain. (**A**) Microscopic investigation of wt and Δku80 grown on BMM medium after 3, 4, 5, and seven days of growth. Ascus rosettes isolated from perithecia were recorded after 9 days of growth. (**B**) Vegetative growth rate was determined after monitored in race tubes. The vegetative growth rate was calculated in mm/d. The standard deviations are represented as error bars. The vegetative growth rate was evaluated from three independent technical replicates performed in triplicates (n=9). Scales bars for A from left to right: 10 µm; 10 µm; 10 µm; 0.5 mm and 100 µm.

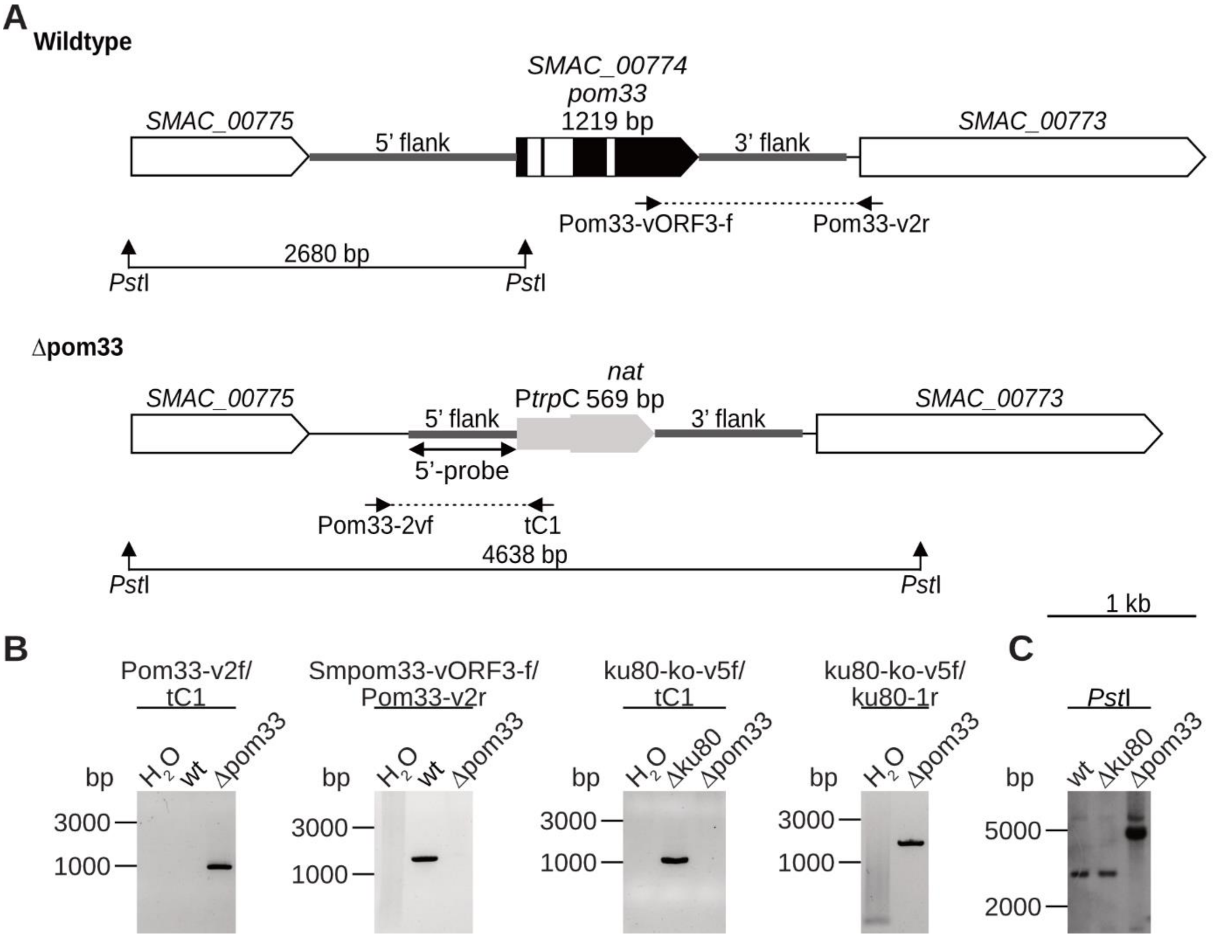

Figure S6: Verification of *Smpom33* deletion by PCR and Southern blot analyses. (**A**) Schematic representation of the *Smpom33* ORF *SMAC_00774* (black arrow) with 3 introns (white boxes). White arrows indicate adjacent ORFs. After Golden-Gate cloning, the *nat* cassette replaced the *Smpom33* ORF. Primer combinations, the corresponding fragment sizes, probe for Southern hybridization and hybridization regions of *Pst*I are indicated. (**B**) PCR verification of the integration of the *nat* cassette into the *pom33* locus. Genomic DNA was isolated from the wt and Δpom33 strain and tested with given primer combinations. Water serves as negative control. (**C**) Confirmation of the *pom33* deletion was performed by Southern blot. The isolated genomic DNA was hydrolyzed with the enzyme *Pst*I. Signals detected correspond to the fragment size of 2680 bp the wt and Δku80 strain and 4638 bp for the *pom33* deletion strain.

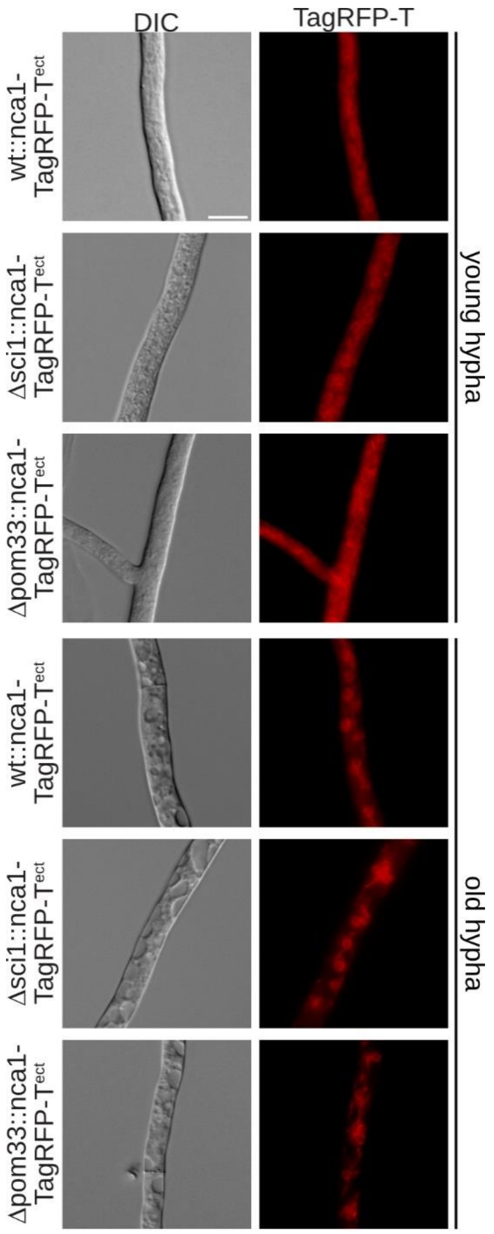

Figure S7: Localization of the ER-marker NCA1-TagRFP-T in the *S. macrospora* wt, Δpom33 and Δsci1 strain. Fluorescence microscopic investigation of the *Tag-RFP-T* tagged ER-marker gene *Smnca1* in the wt, Δpom33 and Δsci1 strain in young and old hyphae. Scale bar = 10 µm. DIC, differential interference contrast.

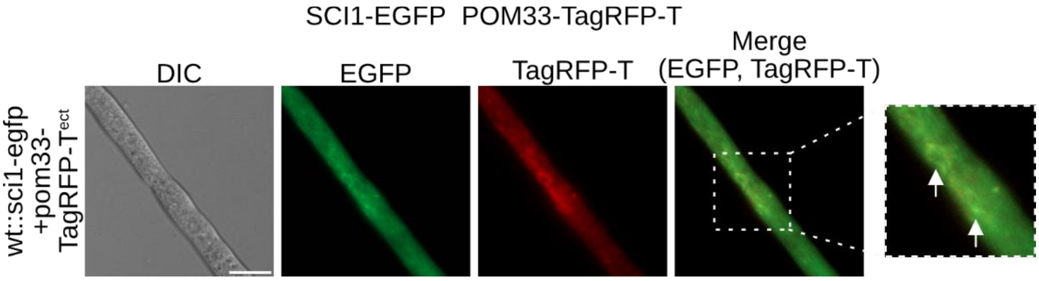

Figure S8: Co-localization of POM33 and SCI1 in *S. macrospora* wt. Fluorescence microscopic investigation of *S. macrospora* wt co-expressing POM33-TagRFP-T and SCI1-EGFP.e nucleus. Partial co-localization of the fusion proteins is indicated by white arrows in the margin. Scale bar = 10 µm. DIC, differential interference contrast. Detail 2-fold enlargements of the merge pictures are indicated with a dashed frame and shown at the right margin.

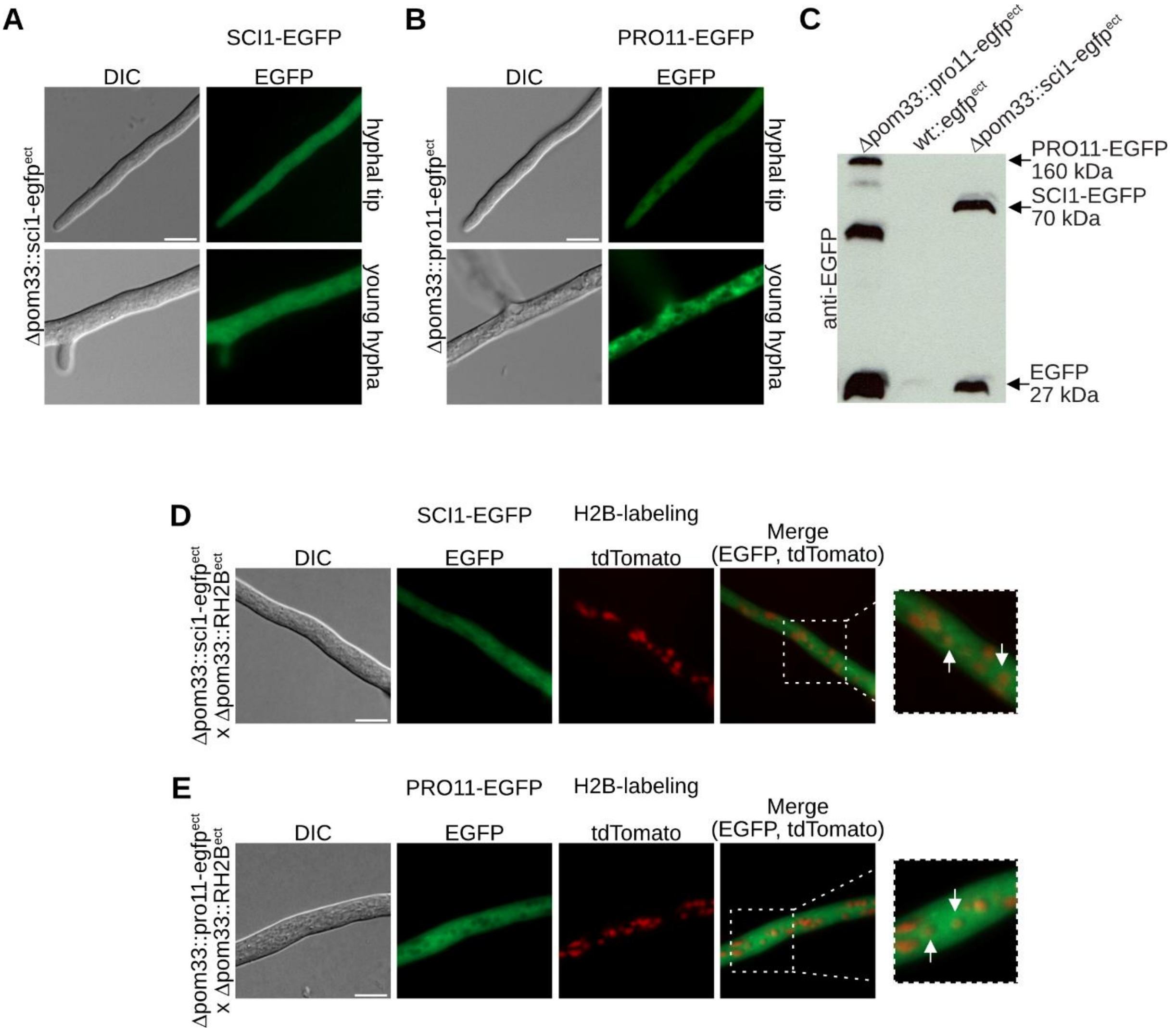

Figure S9: Localization of SCI1 and PRO11 in Δpom33 and Western blot analysis. Fluorescence microscopic investigation of hyphal tips and young hyphae in Δpom33 expressing SCI1-EGFP (**A**) or PRO11-EGFP (**B**). (**C**) Western blot analysis as expression control of the strains, in which wt::egfp^{ect} serves as control. Protein sizes are indicated. Degradation products of PRO11-EGFP are visible. Co-localization of SCI1-EGFP (**D**) or PRO11-EGFP (**E**) with histone 2 B labeled with tdTomato (RH2B) in Δpom33. Partial co-localization is indicated by white arrows in the zoom-in picture. Scale bar = 10 µm. DIC, differential interference contrast. Detail 2-fold enlargements of the merge pictures are indicated with a dashed frame and shown at the right margin.

References

1. Christianson, T.W.; Sikorski, R.S.; Dante, M.; Shero, J.H.; Hieter, P. Multifunctional yeast high-copy-number shuttle vectors. *Gene* **1992**, *110*, 119-122, doi:10.1016/0378-1119(92)90454-w.
2. Carroll, A.M.; Sweigard, J.A.; Valent, B. Improved vectors for selecting resistance to hygromycin. *Fungal Genetics Reports* **1994**, *41*, 22.
3. Klix, V.; Nowrousian, M.; Ringelberg, C.; Loros, J.J.; Dunlap, J.C.; Pöggeler, S. Functional characterization of MAT1-1-specific mating-type genes in the homothallic ascomycete *Sordaria macrospora* provides new insights into essential and nonessential sexual regulators. *Eukaryot Cell* **2010**, *9*, 894-905, doi:10.1128/EC.00019-10.
4. Bloemendal, S.; Bernhards, Y.; Bartho, K.; Dettmann, A.; Voigt, O.; Teichert, I.; Seiler, S.; Wolters, D.A.; Pöggeler, S.; Kück, U. A homologue of the human STRIPAK complex controls sexual development in fungi. *Molecular microbiology* **2012**, *84*, 310-323.
5. Dahlmann, T.A.; Terfehr, D.; Becker, K.; Teichert, I. Golden Gate vectors for efficient gene fusion and gene deletion in diverse filamentous fungi. *Current Genetics* **2020**, 1-14.
6. Pöggeler, S.; Masloff, S.; Hoff, B.; Mayrhofer, S.; Kück, U. Versatile EGFP reporter plasmids for cellular localization of recombinant gene products in filamentous fungi. *Current genetics* **2003**, *43*, 54-61.
7. Nordzieke, S.; Zobel, T.; Franzel, B.; Wolters, D.A.; Kück, U.; Teichert, I. A fungal sarcolemmal membrane-associated protein (SLMAP) homolog plays a fundamental role in development and localizes to the nuclear envelope, endoplasmic reticulum, and mitochondria. *Eukaryot Cell* **2015**, *14*, 345-358, doi:10.1128/EC.00241-14.
8. Werner, A.; Otte, K.; Stahlhut, G.; Hanke, L.M.; Pöggeler, S. The Glyoxysomal Protease LON2 Is Involved in Fruiting-Body Development, Ascosporogenesis and Stress Resistance in *Sordaria macrospora*. *J Fungi (Basel)* **2021**, *7*, 1-17, doi:10.3390/jof7020082.
9. Reschka, E.J.; Nordzieke, S.; Valerius, O.; Braus, G.H.; Pöggeler, S. A novel STRIPAK complex component mediates hyphal fusion and fruiting-body development in filamentous fungi. *Molecular microbiology* **2018**, *110*, 513-532.
10. Oliveros, J.C. VENNY. An interactive tool for comparing lists with Venn Diagrams. **2007-2015**.
11. Blum, M.; Chang, H.Y.; Chuguransky, S.; Grego, T.; Kandasaamy, S.; Mitchell, A.; Nuka, G.; Paysan-Lafosse, T.; Qureshi, M.; Raj, S.; et al. The InterPro protein families and domains database: 20 years on. *Nucleic Acids Res* **2021**, *49*, D344-D354, doi:10.1093/nar/gkaa977.
12. Combet, C.; Blanchet, C.; Geourjon, C.; Deleage, G. NPS@: network protein sequence analysis. *Trends in biochemical sciences* **2000**, *25*, 147-150.
13. Klausen, M.S.; Jespersen, M.C.; Nielsen, H.; Jensen, K.K.; Jurtz, V.I.; Sonderby, C.K.; Sommer, M.O.A.; Winther, O.; Nielsen, M.; Petersen, B.; et al. NetSurfP-2.0: Improved prediction of protein structural features by integrated deep learning. *Proteins* **2019**, *87*, 520-527, doi:10.1002/prot.25674.
14. Chadrin, A.; Hess, B.; San Roman, M.; Gatti, X.; Lombard, B.; Loew, D.; Barral, Y.; Palancade, B.; Doye, V. Pom33, a novel transmembrane nucleoporin required for proper nuclear pore complex distribution. *J Cell Biol* **2010**, *189*, 795-811, doi:10.1083/jcb.200910043.
